# The human milk oligosaccharide 3**′**sialyllactose reduces low-grade inflammation and atherosclerosis development in mice

**DOI:** 10.1172/jci.insight.181329

**Published:** 2024-11-08

**Authors:** Ariane R. Pessentheiner, Nathanael J. Spann, Chloe A. Autran, Tae Gyu Oh, Kaare V. Grunddal, Joanna K.C. Coker, Chelsea D. Painter, Bastian Ramms, Austin W.T. Chiang, Chen-Yi Wang, Jason Hsiao, Yiwen Wang, Anthony Quach, Laela M. Booshehri, Alexandra Hammond, Chiara Tognaccini, Joanna Latasiewicz, Lisa Willemsen, Karsten Zengler, Menno P.J. de Winther, Hal M. Hoffman, Martin Philpott, Adam P. Cribbs, Udo Oppermann, Nathan E. Lewis, Joseph L. Witztum, Ruth Yu, Annette R. Atkins, Michael Downes, Ron M. Evans, Christopher K. Glass, Lars Bode, Philip L.S.M. Gordts

**Affiliations:** 1 Department of Medicine, UCSD, La Jolla, California, USA.; 2 Institute of Molecular Biosciences, University of Graz, Graz, Austria.; 3 Department of Cellular and Molecular Medicine and; 4 Department of Pediatrics at UCSD, La Jolla, California, USA.; 5 Gene Expression Laboratory, Salk Institute for Biological Studies, La Jolla, California, USA.; 6 Department of Bioengineering at UCSD, La Jolla, California, USA.; 7 Novo Nordisk Foundation Center for Biosustainability, La Jolla, California, USA.; 8 Botnar Research Centre, Nuffield Department of Orthopedics, Rheumatology and Musculoskeletal Sciences, NIH Research Oxford Biomedical Research Unit (BRU), and; 9 Oxford Centre for Translational Myeloma Research University of Oxford, Oxford, United Kingdom.; 10 Department of Medical Biochemistry, Experimental Vascular Biology, Amsterdam Cardiovascular Sciences, Amsterdam Infection and Immunity, Amsterdam University Medical Center (UMC), University of Amsterdam, Amsterdam, the Netherlands.; 11 Center for Microbiome Innovation, UCSD, La Jolla, California, USA.; 12 Rady Children’s Hospital of San Diego, San Diego, California, USA.; 13 Larsson-Rosenquist Foundation Mother-Milk-Infant Center of Research Excellence (MOMI CORE) and; 14 Glycobiology Research and Training Center, UCSD, La Jolla, California, USA.

**Keywords:** Cell biology, Inflammation, Atherosclerosis, Epigenetics, Macrophages

## Abstract

Macrophages contribute to the induction and resolution of inflammation and play a central role in chronic low-grade inflammation in cardiovascular diseases caused by atherosclerosis. Human milk oligosaccharides (HMOs) are complex unconjugated glycans unique to human milk that benefit infant health and act as innate immune modulators. Here, we identify the HMO 3′sialyllactose (3′SL) as a natural inhibitor of TLR4-induced low-grade inflammation in macrophages and endothelium. Transcriptome analysis in macrophages revealed that 3′SL attenuates mRNA levels of a selected set of inflammatory genes and promotes the activity of liver X receptor (LXR) and sterol regulatory element binding protein-1 (SREBP1). These acute antiinflammatory effects of 3′SL were associated with reduced histone H3K27 acetylation at a subset of LPS-inducible enhancers distinguished by preferential enrichment for CCCTC-binding factor (CTCF), IFN regulatory factor 2 (IRF2), B cell lymphoma 6 (BCL6), and other transcription factor recognition motifs. In a murine atherosclerosis model, both s.c. and oral administration of 3′SL significantly reduced atherosclerosis development and the associated inflammation. This study provides evidence that 3′SL attenuates inflammation by a transcriptional mechanism to reduce atherosclerosis development in the context of cardiovascular disease.

## Abstract 2

Human milk oligosaccharide 3’sialyllactose inhibits inflammation in macrophages and endothelium, reducing atherosclerosis development, and potentially benefiting cardiovascular health.

## Graphical abstract

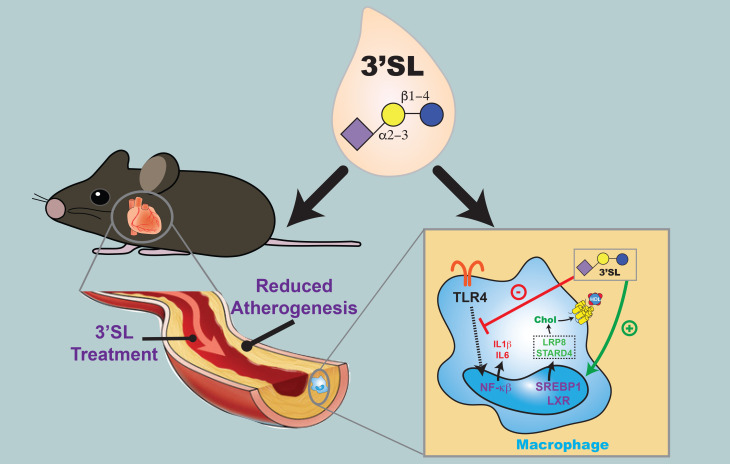

## Introduction

Cardiovascular diseases (CVD) are globally the leading cause of death for both men and women and are mainly caused by complications of atherosclerosis, a complex chronic inflammatory disorder. Atherosclerosis initiates when circulating low-density lipoproteins (LDL) are trapped in the subendothelial extracellular matrix of arteries where they get modified into oxidized LDL (oxLDL). oxLDL activates the overlying endothelium, promoting infiltration of monocytes that differentiate into macrophages, which internalize the oxLDL and turn into cholesterol-loaded foam cells. The retained foam cells promote a chronic inflammatory response and induce proinflammatory cytokine secretion such as IL-1b, IL-6, TNF, and antiinflammatory cytokines among others. Many preclinical studies provide evidence that lowering systemic inflammation or promoting inflammation resolution would promote prevention of major adverse cardiovascular events (MACE) (1–3). The Canakinumab Anti-Inflammatory Thrombosis Outcomes Study (CANTOS) trial was the first large-scale proof-of-concept trial testing quarterly administered canakinumab, a neutralizing IL-1β monoclonal antibody, to a very high-risk cohort of patients with postmyocardial infarction who have reduced LDL-cholesterol and elevated high-sensitivity C-reactive protein (hsCRP) levels. Despite the promising results, the adverse effects, very high costs, and administration via injections warrant searches for additional novel, safe, and effective therapeutics targeting chronic inflammation that will benefit a greater population of patients with CVD.

Human milk oligosaccharides (HMOs) are a natural and abundant component of human milk that have a variety of biological functions shown to promote development and regulate immune function (4–6). All HMOs are unconjugated glycans that carry lactose at their reducing end. Lactose can either be fucosylated to yield fucosyllactoses, sialylated to yield sialyllactoses, or elongated and branched to yield a total of more than 150 distinct oligosaccharides, each with potentially different structure-dependent activity profiles. Unlike most oligosaccharides, such as lactose, HMOs resist the low pH in the stomach as well as digestion by pancreatic and brush border enzymes (4, 7, 8). Approximately 1% of the ingested HMO amount is absorbed, reaches the systemic circulation (9, 10), and is excreted in the urine (11–13). Originally, HMOs were considered prebiotics that help shape the gut microbiome with health benefits for the breastfed infant. It has become increasingly clear that HMOs also have beneficial immunomodulatory properties independent of the gut microbiome (14, 15). HMOs act locally on cells of the mucosa-associated lymphoid tissues, and absorbed HMOs can act on a systemic level. Most reports attribute antiinflammatory properties to HMOs. However, the effects of HMOs and their mechanism of action remain uncharacterized in the majority of low-grade chronic inflammatory diseases in adults such as atherosclerosis.

In this study, we tested the hypothesis that a specific HMO attenuates low-grade macrophage inflammation and atherosclerosis development in mice. Using an unbiased screen, we identified 1 specific HMO called 3′sialyllactose (3′SL) that was most effective in reducing macrophage inflammation while other, structurally distinct HMOs had no effect. 3′SL significantly reduced IL-1β IL-6, IL-10, and TNF expression in LPS-activated macrophages, in both cell lines and primary cells of both murine and human origin. Mechanistically, we provide evidence that 3′SL also reduces inflammation by influencing the recruitment of liver X receptor/sterol regulatory element binding protein(LXR/SREBP) to enhancers of enzymes involved in production of resolving lipids. Importantly, our study shows that 3′SL administration reduced the development of atherosclerotic lesions in a preclinical murine model.

## Results

### HMOs attenuate low-grade inflammation.

**Figure 1:**
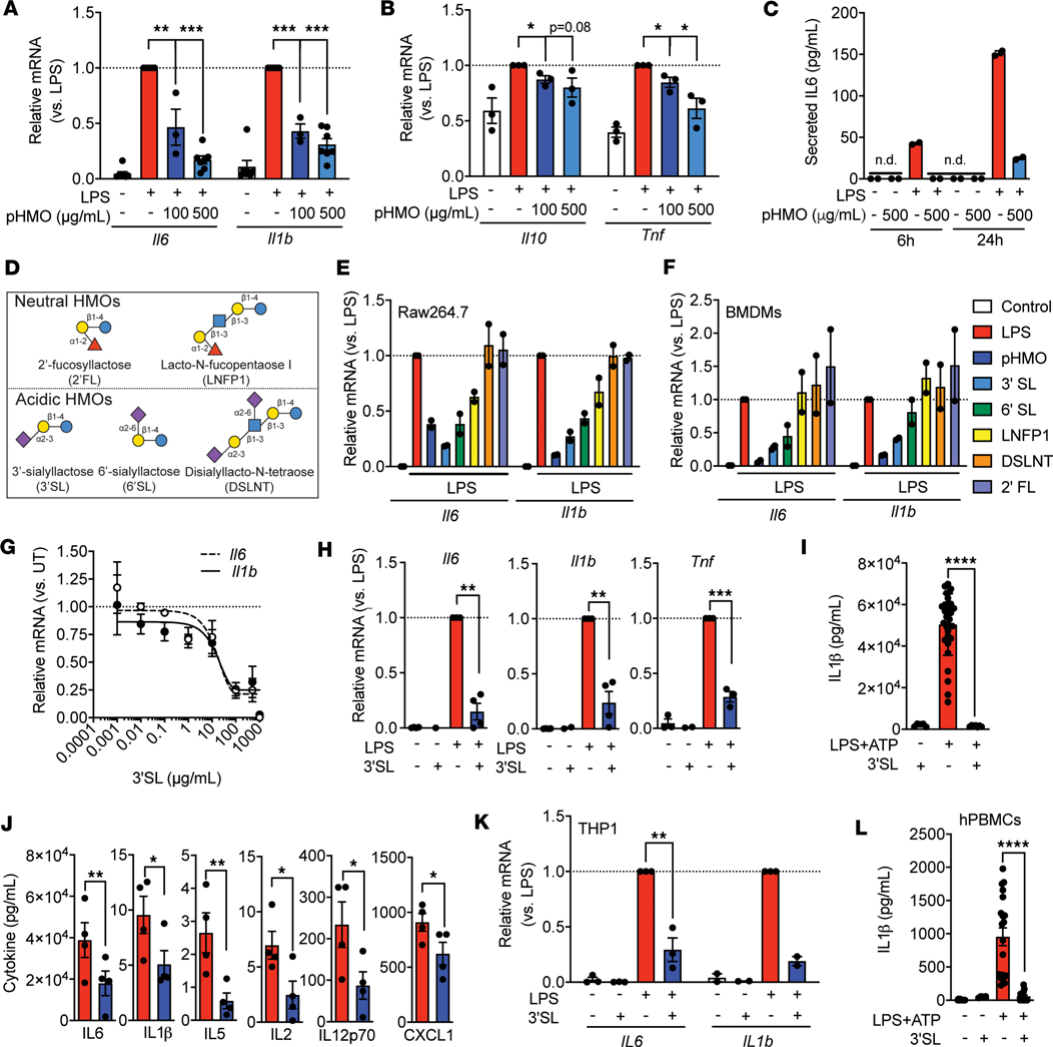
HMOs, particularly 3′SL, reduce inflammatory cytokine expression in LPS-activated murine macrophages and human monocytes. (**A** and **B**) Relative mRNA levels of *Il6* and *Il1b* (**A**), and *Il10* and *Tnf* (**B**) in Raw264.7 cells with LPS ± pooled HMO (pHMO) incubation (*n* = 2). (**C**) IL-6 protein release 6 and 24 hours after LPS ± pHMO incubation (*n* = 3). (**D**) Depiction of individually used HMOs. (**E** and **F**) Relative mRNA levels of *Il6* and *Il1b* in Raw 264.7 (**E**) and murine bone marrow–derived macrophages (BMDMs) (**F**) when treated with individual HMOs (*n* = 2). (**G**) Dose-response curve of 3′SL in BMDMs (*n* = 3). (**H**) Relative mRNA levels of *Il6*, *Il1b*, and *Tnf* expression in PBS, 3′SL, or LPS ± 3′SL (100 μg/mL) treatment (*n* = 3–4). (**I**) IL-1β protein release for 24 hours in BMDMs after LPS+ATP coincubation ± 3′SL (*n* ≥ 5). (**J**) Cytokine concentrations in the conditioned medium of BMDMs with 24 hours of LPS ± 3′SL incubation (*n* = 4). (**K**) Relative mRNA levels of *IL6* and *IL1b* in LPS-activated human THP-1 cells treated ± 3′SL (*n* = 3). (**L**) IL-1β protein release 24 hours after LPS+ATP coincubation ± 3′SL (100 μg/mL) in human peripheral blood monocytes (hPBMCs) from 3 healthy donors. If not otherwise stated, incubations lasted for 6 hours with 10 ng/mL LPS and 100 μg/mL 3′SL or above indicated concentrations of HMOs. Two-way ANOVA with Fisher’s LSD test. *P < 0.05; **P < 0.01; ***P < 0.001; ****P < 0.0001. Data represented as mean ± SEM.

Several studies support immunomodulatory properties of HMOs in different disease models. We determined the potential of HMOs to attenuate low-grade inflammation relevant for atherosclerosis in the RAW 264.7 murine macrophage cell line. To test this, we measured IL-6, IL-10, and TNF production in RAW 264.7 macrophages stimulated with or without a low-grade dose (10 ng/mL) of the TLR4 activator LPS (16, 17). We cotreated the RAW 264.7 macrophages with PBS or a low (100 μg/mL) or high (500 μg/mL) dose of LPS-free HMOs pooled from different donors (pHMOs) to capture the entire variety and chemical space of HMOs that vary between individual women (Figure 1, A and B). Coincubation of LPS with pHMO significantly reduced mRNA levels of proinflammatory cytokines *Il1b*, *Il6*, *Il10*, and *Tnf* in a dose-dependent manner compared with LPS control (Figure 1, A and B). The most potent inhibition was observed at the higher pHMO concentration (500 μg/mL) resulting in ~70% and ~80% reduction in LPS-induced *Il1b* and *Il6* mRNA levels, respectively, compared with LPS alone. This translated in a ~80% reduction in IL-6 protein secretion after 6 and 24 hours (Figure 1C). The results confirm that pooled HMOs can attenuate low-grade inflammation in macrophages.

### 3′SL inhibits inflammation in human and murine macrophages.

Next, we attempted to identify the most potent specific HMO responsible for attenuating LPS-induced inflammation in RAW 264.7 macrophages. First, we applied anion exchange chromatography to fractionate pHMO by charge into neutral (nonsialylated) and acidic (sialylated) HMOs. RAW 264.7 macrophages were cotreated with LPS (10 ng/mL) and either neutral HMOs, acidic HMOs, or lactose (each 500 μg/mL) (Supplemental Figure 1, A and B; supplemental material available online with this article; https://doi.org/10.1172/jci.insight.181329DS1). Lactose did not affect inflammatory cytokine mRNA levels in RAW 264.7 macrophages (Supplemental Figure 1, A and B). Both predominantly acidic and neutral pHMO fractions were able to significantly decrease LPS-induced mRNA levels of *Il1b* and *Il6* (Supplemental Figure 1, A and B). However, crude neutral HMOs contained a small amount of the acidic HMO, 3′SL, and acidic HMOs contained small amounts of lactose and the 2 neutral HMOs, 2′fucosyllactose (2′FL) and difucosyllacto-*N*-hexaose (DFLNH) (Supplemental Figure 1, B and C). Therefore, we tested LPS-free pHMO (500 μg/mL) alongside individual HMOs from both subfractions — namely 2′FL, disialyllacto-*N*-tetraose (DSLNT), lacto-*N*-fucopentaose I (LNFP I), 3′SL, and 6′SL — each at a concentration of 100 μg/mL (Figure 1D). The 2 sialylated HMOs, 3′SL and 6′SL, had the strongest *Il6* and *Il1b* inhibition in LPS-activated macrophages, while fucosylated 2′FL did not exhibit antiinflammatory properties (Figure 1E). Importantly, 3′SL-mediated antiinflammatory effects observed in RAW 264.7 macrophages were confirmed in murine primary bone marrow–derived macrophages (BMDMs) (Figure 1F). Following these results, we identified 3′SL as the most potent HMO. Dose-range–finding studies in BMDMs identified IC_50_ values for 3′SL around 15 μg/mL (Figure 1G), which is comparable with 3′SL plasma concentrations in breastfed infants (9, 10). Importantly, in the absence of LPS, incubation with 3′SL (100 μg/mL) did not affect cytokine mRNA levels compared with PBS (Figure 1H). The inhibition of IL-6, IL-10, IL-12p70, IL-2, IL-5, and IL-1β was confirmed on the protein level in culture medium of LPS-activated BMDMs cotreated with 3′SL for 24 hours, while Keratinocyte Chemoattractant-Growth-Regulated Oncogene (KC-GRO) was unaffected (Figure 1, I and J). Importantly, the antiinflammatory effects of 3′SL translated to human immune cells, as 3′SL also attenuated *IL6* and *IL1b* mRNA levels in LPS-stimulated THP1 human monocytic cells (Figure 1K), and it attenuated protein secretion of IL-1β in human peripheral blood monocytes (hPBMCs) (Figure 1L). Taken together, we identified that the HMO 3′SL effectively reduced low-grade inflammatory cytokine production in murine and human macrophages and monocytes stimulated with LPS.

### 3′SL does not alter TLR4 activation and NF-κB signaling.

**Figure 2:**
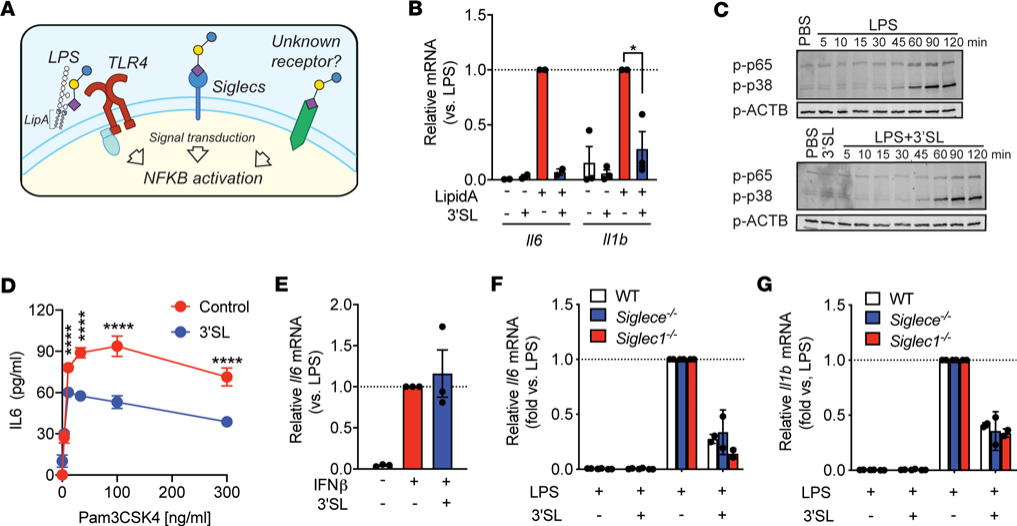
3′SL does not engage with carbohydrate part of LPS, neither reduce NF-κB signaling, nor IFN-γ signaling and Siglec interactions. (**A**) Scheme of potential 3′SL effector pathways. (**B**) Relative *Il6* and *Il1b* mRNA levels in lipid A–activated BMDMs treated ± 3′SL. (**C**) Western blot of p-p65 and p-p38 after different times of LPS ± 3′SL stimulation. One representative blot of *n* = 3. (**D**) IL-6 concentrations in the conditioned medium of BMDMs with 6 hours Pam3CSK4 (at indicated doses) ± 3′SL incubation (*n* = 4). (**E**) Relative *Il6* and *Il1b* mRNA levels in BMDMs stimulated with 10 ng/mL IFN-β INF-β ± 3′SL. (**F** and **G**) Relative *Il6* and *Il1b* mRNA levels in *Siglec1^–/–^* (**F**) and *SiglecE^–/–^* (**G**) BMDMs stimulated with LPS ± 3′SL. All stimulations 10 ng/mL LPS, 100 μg/mL 3′SL. Two-way ANOVA with Fisher’s LSD test. *P < 0.05; ****P < 0.0001). Data represent mean ± SEM (*n* = 2–3 of individually isolated BMDMs).

To gain insight into the potential mechanism involved in 3′SL-mediated reduction of proinflammatory cytokines, we explored several possible cell-surface interactors (Figure 2A). First, we tested whether 3′SL directly inhibits LPS binding to the TLR4 receptor through interaction with the carbohydrate moiety of LPS (16, 17). Therefore, we activated BMDMs with the lipid portion of LPS (lipid A). Activation of *Il6* and *Il1b* mRNA levels was comparable with LPS, and 3′SL attenuated the expression of both cytokines in the presence of lipid A to the same extent as LPS by ~90% and ~70%, respectively (Figure 2B). The results suggest that 3′SL attenuates LPS-driven inflammation without inhibiting carbohydrate interactions. Further Western blot analysis revealed that the time-dependent phosphorylation of the key signaling molecules NF-κB (p-p65) and mitogen-activated protein kinases (MAPK, p-p38) pathways were not altered by 3′SL coincubation with LPS (Figure 2C), suggesting that 3′SL does not directly impact the TLR4-mediated signaling cascade. Furthermore, IL-6 production in BMDMs after stimulation with the TLR2 agonist, Pam3CSK4, was also significantly attenuated 1.5-fold by 3′SL coincubation (Figure 2D). Upon LPS-activation in macrophages, the TLR4/MD2 complex becomes internalized into endosomes and triggers signaling cascades that activate the expression of type I IFN such as IFN-β (18). IFNs are critical for innate immune responses (18, 19) and implicated in the pathogenesis of chronic inflammatory diseases such as atherosclerosis (20–22). Therefore, we tested if 3′SL also alleviates IFN-mediated inflammation. Exogenous stimulation of BMDMs with IFN-β induced *Il6* but not *Il1b* mRNA levels (Figure 2E). Coincubation of IFN-β with 3′SL did not attenuate *Il6* mRNA levels, indicating that antiinflammatory effects of 3′SL are not mediated by attenuating the downstream IFN-β–dependent pathway induced by TLR4 activation. Thus, 3′SL attenuates inflammatory gene expression in TLR4-activated macrophages without affecting TLR4 induction and NF-κB signaling.

### Antiinflammatory properties of 3′SL are independent of sialic acid binding siglec receptors.

There are 2 main sialic acid–binding transmembrane receptors presented at the cell surface of murine macrophages, Siglec1 and SiglecE (Supplemental Figure 2). These receptors in particular bind terminal sialic acids to modulate inflammatory signaling via intracellular tyrosine–based signaling motifs, especially immunoreceptor tyrosine–based inhibitory motifs (ITIMs) that are implicated in cell signaling and endocytosis (23, 24). SiglecE preferentially interacts with sialic acid linked in the α2-3 position to D-galactose, such as 3′SL. Sialoadhesin (Siglec1) shares the substrate specificity with SiglecE and has been shown to also bind 3′SL (23, 25). As 3′SL features a sialic acid at the nonreducing end of the lactose backbone, we tested these known sialic acid–binding receptors as potential target receptors on macrophages that mediate the antiinflammatory actions of 3′SL (Figure 2, F and G). We initially probed the importance of SiglecE, since Siglec1 lacks the ITIM required to attenuate inflammation. BMDMs derived from *Siglece*-deficient (*Siglece^–/–^*) mice were stimulated with PBS or 3′SL in the presence or absence of LPS. Similar as in WT BMDMs, 3′SL significantly reduced LPS-induced *Il6* and *Il1b* mRNA levels by 74% and 65%, respectively (Figure 2, F and G). Analogous results were seen using BMDMs derived from Sialoadhesin-KO mice (*Siglec1^–/–^*), providing evidence against a direct involvement of SiglecE and Siglec1 in the antiinflammatory effects of 3′SL (Figure 2, F and G). Taken together, the results suggest that 3′SL does not evoke its action via carbohydrate interactions with LPS, altering the TLR4 signaling cascade, or binding to the antiinflammatory Siglec receptors.

### 3′SL represses and induces a selected group of LPS-responsive inflammatory genes.

**Figure 3:**
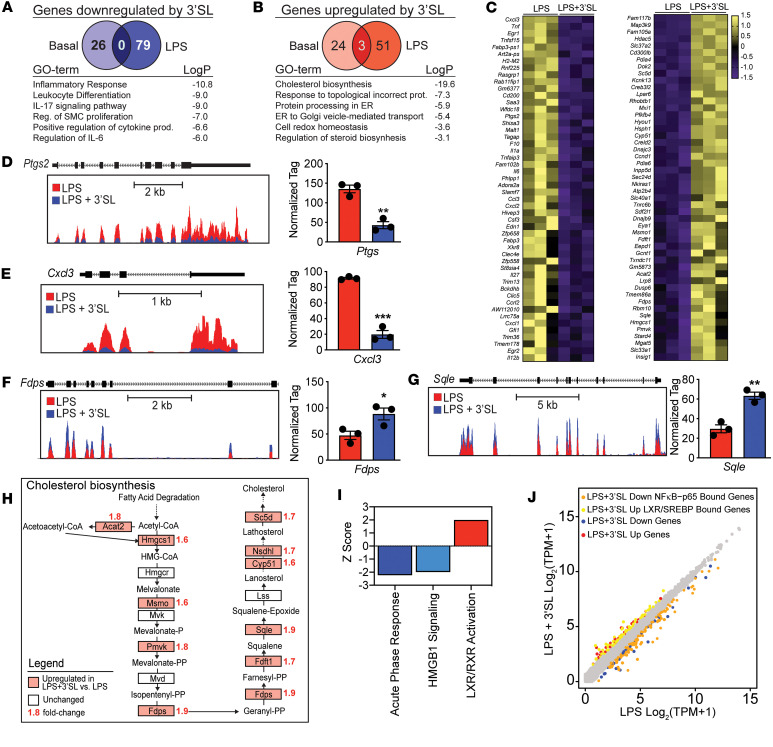
3′SL downregulates inflammatory pathways and upregulates cholesterol biosynthesis in LPS-stimulated BMDMs. (**A** and **B**) Venn diagrams of differentially expressed mRNAs of genes detected by RNA-Seq (cut-off *P* < 0.05 and fold change [FC] > 1.5) of 3′SL downregulated (**A**) and upregulated (**B**) mRNA levels of genes in LPS-activated BMDMs compared with quiescent, PBS-treated BMDMs. Corresponding pathway analyses of LPS versus LPS + 3′SL–treated BMDMs are below the diagrams (*n* = 3). (**C**) Heatmaps representing *Z*-normalized row mRNA levels of each gene for RNA-Seq from independent biological duplicates showing the 50 most upregulated (right) or downregulated (left) genes in BMDMs at 6 hours after LPS ± 3′SL stimulation. (**D** and **E**) UCSC genome browser images illustrating normalized tag counts for *Ptgs2* and *Cxcl3* with normalized tag count averages (*n* = 3). (**F** and **G**) UCSC genome browser images illustrating relative mRNA levels for cholesterol biosynthesis genes *Fdps* and *Sle* with normalized tag count averages (*n* = 3). (**H**) Scheme of KEGG-pathway cholesterol biosynthesis. Red marked genes are upregulated by 3′SL coincubation with LPS. Red numbers above gene names indicate fold-change. (**I**) Enriched pathways identified by Ingenuity Pathway Analysis (IPA) software. All significantly, differentially expressed mRNAs of genes in the LPS versus LPS + 3′SL RNA-Seq datasets were used for the analysis with IPA (cut-off > 1.5-fold) with *Z* score ≥ ±2. (**J**) Scatter plot depicting the relationship between fold change of 3′SL and LPS–repressed and 3′SL-induced mRNAs of genes, overlaid with LXR- and SREBP-associated accessible loci from a previously generated ATAC-Seq dataset (33). Unpaired 2-tailed Student’s *t* test. *P < 0.05; **P < 0.01; ***P < 0.001. Data represent mean ± SEM.

To investigate 3′SL-induced changes in macrophage gene expression throughout the course of an inflammatory response, we subjected BMDMs treated for 6 hours with or without (10 ng/mL) LPS in the presence or absence of (100 μg/mL) 3′SL to whole transcriptome (RNA-Seq) analysis. RNA-Seq results show that using a 1.5-fold difference and FDR < 0.05, 3′SL altered the mRNA levels of 133 genes under LPS stimulation and 53 genes under basal conditions (Figure 3, A and B). In contrast, LPS activation induced changes in more than 5,000 genes using these same criteria (Supplemental Figure 3A). The subset of the 79 genes downregulated by 3′SL under LPS stimulation are categorized by gene ontology analysis in the NF-κB Inflammatory response (Figure 3, A and C). We define this subset of 3′SL-regulated genes as 3′SL inflammatory repressed genes that include *Il6* and *Tnf* and inflammatory markers, such as endothelin 1 (*Edn1*), chemokine C-X-C motif ligand 1-3 (*Cxcl1-3)*, prostaglandin endoperoxide synthase 2 (*Ptgs2*, also known as *Cox2*), and the inflammatory mediator serum amyloid A3 (SAA3) (Figure 3C) (26–28). The exonic distribution of normalized tag counts for representative genes is illustrated in Figure 3, D and E, and Supplemental Figure 3, D and E; qPCR further confirms attenuated gene expression of inflammatory marker genes (Supplemental Figure 3). The 3′SL inflammatory repressed genes do not overlap with genes downregulated in BMDMs under basal noninflammatory conditions (Supplemental Figure 3, A–C). However, this latter 3′SL downregulated basal gene set also includes inflammation annotated genes such as fatty acid binding protein 4 (*Fabp4*), PYD and CARD domain containing (*Pycard*), C1q and tumor necrosis factor–related 12 (*C1qtnf12*), and Cd180 (Supplemental Figure 3, B and C). The observations suggest that 3′SL does not result in panattenuation of TLR4-induced changes in gene expression but, rather, represses a specific subset of proinflammatory genes.

### Sterol and fatty acid metabolism genes are upregulated by 3′SL during inflammation.

During LPS stimulation, 3′SL induced an almost equal number of genes compared with the downregulated group of genes. The majority of the 54 upregulated genes are involved in sterol biosynthesis and nuclear receptor activation as well as ER-Golgi transport and ER protein processing (Figure 3B). This subset of 3′SL inflammatory–induced genes has a minimal overlap with genes upregulated under basal conditions, indicating an inflammation-dependent context wherein 3′SL affects these 3′SL inflammatory–induced genes (Figure 3B and Supplemental Figure 3, A–C). Interestingly, among the 3′SL inflammatory–induced genes, a significant portion are involved in cholesterol biosynthesis, such as 3-hydroxy-3-methylglutaryl-CoA synthase 1 (*Hmgcs1*), farnesyl diphosphate synthase (*Fdps*), and squalene monooxygenase (*Sqle*), and fatty acid metabolism, such as Acyl-CoA desaturase 2 (*Scd2*) and acetyl-CoA acetyltransferase 2 (*Acat2*) (Figure 3, C–G, and Supplemental Figure 3G). Another 3′SL inflammatory–induced gene is StAR-related lipid transfer protein 4, which is important for regulation of cholesterol homeostasis and membrane trafficking (29) (Figure 3H and Supplemental Figure 3, H and I). LDL receptor–related protein 8 (*Lrp8*) was the most upregulated 3′SL inflammatory–induced gene and was also upregulated by 3′SL in quiescent BMDMs (Figure 3, C and F, and Supplemental Figure 3, G–I). *Lrp8* encodes for an apolipoprotein E (apoE) receptor and promotes cholesterol efflux and lipoprotein clearance to exert its antiatherogenic effects (30–32). These results support that 3′SL has a selective effect on gene expression changes during LPS stimulation. Importantly, the data also suggest that 3′SL engages transcription factors affecting genes involved in cholesterol and fatty acid homeostasis.

### The 3′SL response is associated with activation of LXR and SREBP signal –dependent transcription factors.

Cholesterol and fatty acid metabolism genes are transcriptionally regulated by the nuclear LXRs, SREBP1, and SREBP2. Ingenuity Pathway Analysis (IPA) of the RNA-Seq results revealed 3 metabolic pathways enriched or repressed by 3′SL (*Z* score of > 2 or < –2, respectively). Importantly, it independently identified LXR/retinoid X receptor–regulated (LXR/RXR-regulated) pathways as the most significantly enriched (Figure 3I). Importantly, *Lxra* (*Nr1r3* gene) and *Lxrb* (*Nr1h2* gene) mRNA levels were not influenced by 3′SL incubation (Supplemental Figure 3J). We used previously generated ATAC-Seq data from BMDMs (33) to assign nearby putative regulatory enhancer regions to 3′SL regulated genes, as defined by accessible regions within 20 Kb of the gene body. To assess 3′SL-mediated modulation’s potential via LXR and SREBP, we overlaid these associated accessible loci of the 3′SL inflammation–induced genes with ChIP-Seq data for LXR and SREBP obtained in BMDMs after stimulation with the LXR/SREBP agonist GW3965. In alignment with the IPA, we observed that 67% of the 3′SL inflammatory–induced genes are occupied by either LXR and SREBP (Figure 3J), with the majority of these loci cobound by LXR and SREBP. These data suggest a role for LXR and SREBP in the 3′SL-mediated upregulation of lipid metabolism–related genes.

### LXR and SREBP transcriptional activity is modulated by 3′SL.

**Figure 4:**
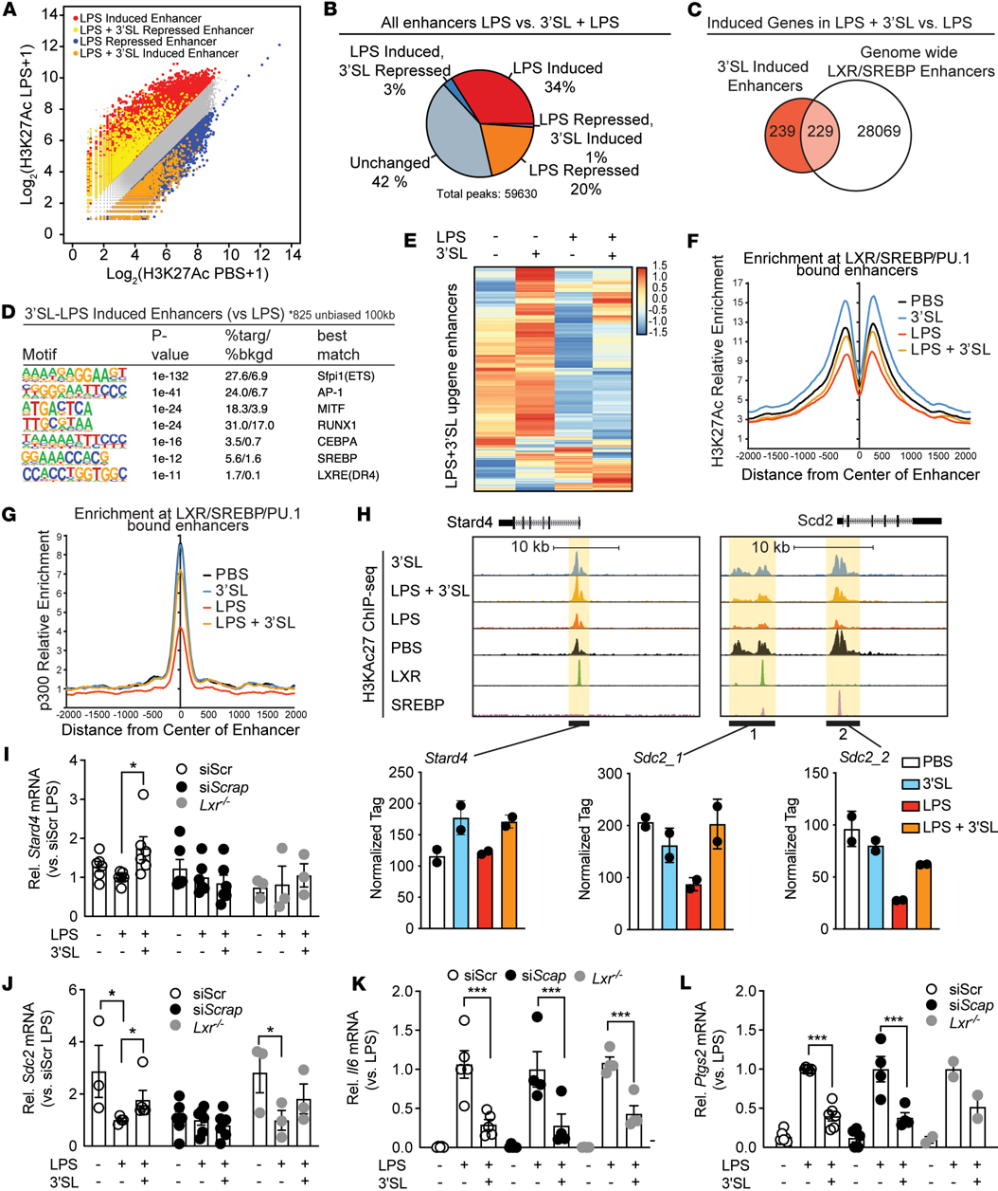
The 3′SL inflammation response is associated with LXR and SREBP signal–dependent transcription factors. (**A**) Scatter plot depicting the enhancers as defined by H3K27ac in their relationship to LPS stimulation with and without 3′SL coincubation. (**B**) Venn-diagram of global enhancers affected by 3′SL and LPS compared with LPS induced enhancers. (**C**) Venn-diagram of 3′SL-LPS–induced genes compared with LPS-induced enhancers associated with LXR/SREBP-bound loci. (**D**) De novo motif analysis of 3′SL-LPS–induced enhancers (versus LPS) using a GC-matched genomic background. (**E**) Heatmap of the fold change in 3′SL upregulated H3K27ac levels at ATAC-Seq–defined gene loci that demonstrated binding for LXR or SREBP. (**F**) Distribution of H3K27Ac tag densities, in the vicinity of genomic regions cobound by LXR, SREBP, or PU.1, in BMDMs treated with indicated stimuli for 6 hours. (**G**) Distribution of p300 tag densities, in the vicinity of genomic regions cobound by LXR, SREBP, or PU.1, in BMDMs treated with indicated stimuli for 6 hours. (**H**) UCSC genome browser images illustrating normalized tag counts for H3K27ac at *Stard4* and *Scd2* target loci together with mapped LXR and SREBP binding sites. (**I**–**L**) Relative mRNA levels of target genes in BMDMs transfected with nontargeting control siRNA (siScr) or si*Scap* or BMDMs isolated from *Lxr*α*/*β-KO mice (*Lxr*^–/–^) stimulated with PBS and LPS ± 3′SL. (*n* = 4 silencing experiments, *Lxr*^–/–^ BMDMs from 3 individual mice). Two-way ANOVA with Fisher’s LSD test. *P < 0.05; **P < 0.01; ***P < 0.001. Data represent mean ± SEM.

Previous studies support a role for LXR and SREBP responsive genes in the resolution phase of TLR-mediated inflammation (33). To investigate the potential of 3′SL in modulating these antiinflammatory genes’ expression at the level of transcription, we probed the associated enhancer landscape by performing ChIP-Seq for accepted markers of transcriptional activity such as histone 3 lysine 27 acetylation (H3K27ac) and p300, a histone acetyltransferase (HAT) in LPS-treated BMDMs with or without 3′SL. H3K27 acetylation is deposited by HATs, such as p300, which are associated with transcriptional coactivators and are highly correlated with regulatory element activity (34). To associate the H3K27ac signal with specific regulatory elements associated with 3′SL-modulated genes, we overlapped H3K27ac with locus-specific ATAC-Seq peaks. More specifically, we first determined H3K27ac levels at ATAC-Seq–defined regions of 3′SL-regulated gene loci (Figure 4A). In line with the RNA-Seq data, we observed that activity at a limited subset of the LPS-induced enhancers were upregulated by 3′SL coincubation (Figure 4B). De novo motif analysis using a GC-matched genomic background shows that these 3′SL inflammatory–induced genes were enriched for myeloid-specific lineage determining transcription factors (LDTF), such as AP-1, RUNX1, CEBPA, and E26 transcription–specific factor (ETS-factor) motifs, but these regions were also specifically enriched for downstream signal-dependent transcription factors (SDTF) such as SREBP and LXR response elements (LXRE) (Figure 4, C and D). For 3′SL upregulated gene loci, we focused on transcriptionally accessible gene loci that demonstrated binding for LXR or SREBP (Figure 4E). H3K27ac levels were robustly induced by 3′SL at those enhancer sites (Figure 4, E–H), as exemplified by genome browser tracks of 3′SL-activated genes such as *Lrp8*, *Fdps*, *Stard4*, and *Sdc2* (Figure 4H and Supplemental Figure 4A). Interestingly, 3′SL treatment alone allowed for significant induction of H3K27ac levels at those LXR/SREBP enhancers (Figure 4, E–H). The data indicate that 3′SL can blunt the LPS repression of LXR/SREBP-regulated genes. This result suggests that 3′SL is conferring activation of these enhancers by modulating the activity of LXR and SREBP transcription factors. This concept is further supported by 3′SL-dependent recruitment of p300 at the LXR/SREBP enhancers (Figure 4G). The LXR/SREBP-bound and 3′SL-induced enhancers are direct targets as demonstrated by p300, SREBP, and LXR recruitment in BMDMs to those sites upon treatment with GW3965 (Supplemental Figure 4, B–E). The observation further supports the idea that 3′SL is a bona fide modulator of SREBP and LXR transcriptional activity.

Next, we set out to test if genetic inactivation of LXR or SREBP prevents the induction and repression of 3′SL inflammation–modulated genes. We targeted SREBP activation by siRNA-mediated knockdown of its upstream regulator SCAP1 (siSCAP; Supplemental Figure 4F) (35, 36). To probe the importance of LXR, we isolated BMDMs from LXRα and LXRβ double-KO mice (*Lxr^–/–^*). As expected, inactivation of LXR and SREBP blunted the induction of 3′SL inflammation–induced genes, such as *Stard4* and *Scd2* (Figure 4, I and J). In contrast, SREBP and LXR targeting did not affect the mRNA levels of 3′SL inflammation–repressed genes, since 3′SL was still able to attenuate LPS-induced mRNA levels of *Il6*, *Ptgs2*, *Il1b*, and *Saa3* equally in LXR-KO and siSCAP treated BMDMs (Figure 4, K and L, and Supplemental Figure 4, G and H). The observation supports the idea that 3′SL is a molecule capable of stimulating SREBP and LXR transcriptional activity in macrophages.

### 3′SL inhibits activation of a subset of TLR4 responsive enhancers.

**Figure 5:**
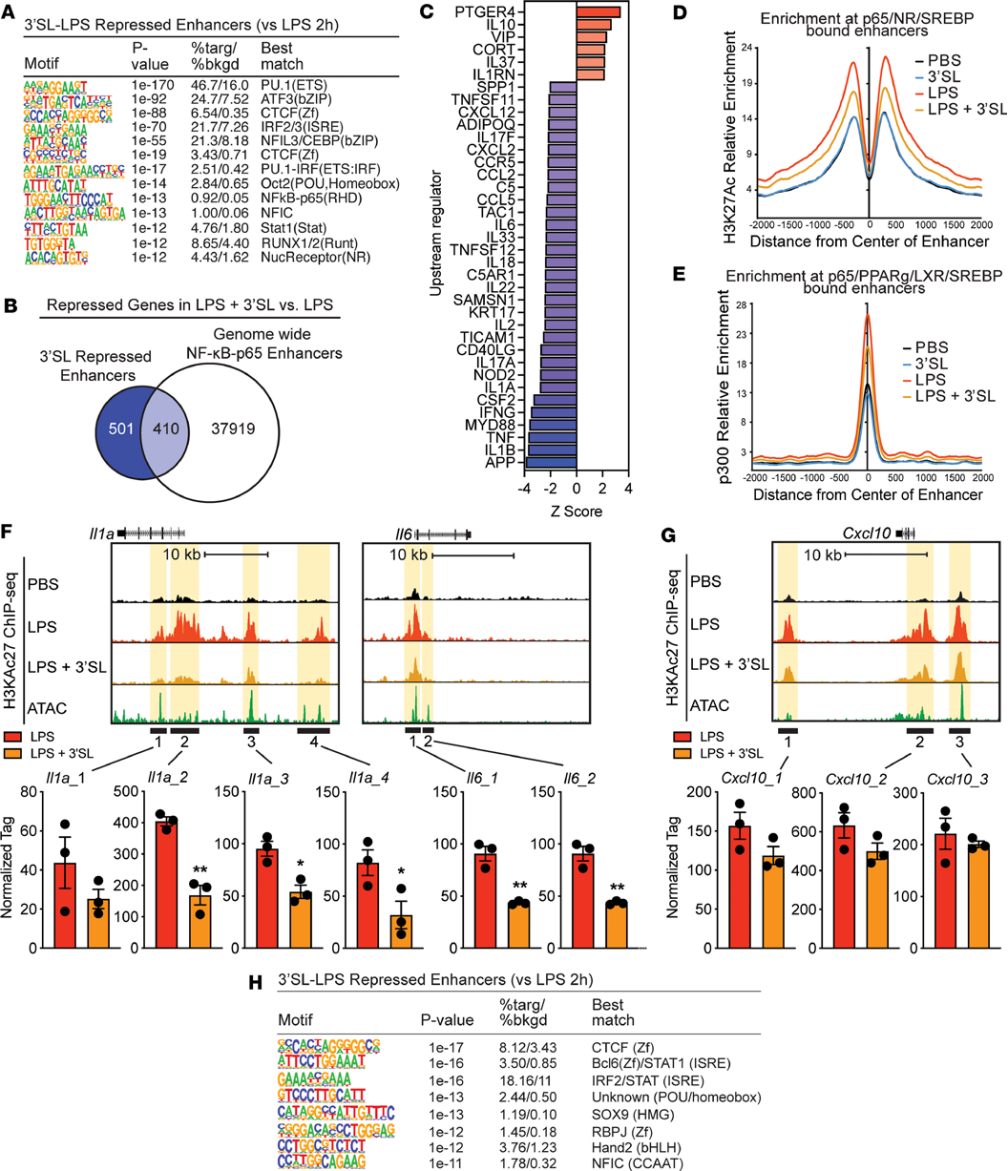
3′SL mediates reprogramming of the NF-κB enhancer landscape activity to attenuate inflammation. (**A**) De novo motif analysis of 3′SL + LPS repressed enhancers (versus LPS) using a GC-matched genomic background. (**B**) Venn diagram of 3′SL-LPS repressed genes compared with LPS induced enhancers associated with NF-κB/p65 bound loci. (**C**) IPA analysis of upstream regulators of differentially expressed genes (|*Z* score| > ± 2). (**D**) Distribution of H3K27ac tag densities in the vicinity of genomic regions cobound by NF-κB/p65 in BMDMs treated with indicated stimuli for 6 hours. (**E**) Distribution of p300 tag densities in the vicinity of genomic regions cobound by NF-κB/p65 in BMDMs treated with indicated stimuli for 6 hours. (**F** and **G**) UCSC genome browser images illustrating normalized tag counts for H3K27ac at *Il1a*, *Il6*,and *Cxcl10* target loci together with mapped ATAC-Seq tags. (**H**) De novo motif analysis of 3′SL + LPS repressed enhancers (versus LPS) using all LPS-induced ATAC-Seq peaks as a background. Unpaired 2-tailed Student’s *t* test. *P < 0.05; **P < 0.01. Data represent mean ± SEM.

Genes negatively affected by 3′SL belong to inflammatory pathways such as the acute phase response and the proinflammatory high mobility group box 1 (HMGB1) signaling (Figure 3I) according to pathway analysis of the RNA-Seq data. We assessed the extent to which altered transcriptional regulation contributes to the 3′SL-mediated repression of inflammatory gene expression. Utilizing H3K27ac ChIP-Seq data, we evaluated the effect of 3′SL on ATAC-Seq and PU.1-bound defined enhancers activated by LPS (Figure 4A). In line with the RNA-Seq data, we observed that activity at a limited subset of the LPS-induced enhancers was negatively affected by 3′SL coincubation (Figure 4B). De novo motif analysis using a GC-matched genomic background showed that these 3′SL-repressed regions demonstrate motif enrichment for the expected macrophage lineage–determining factors (PU.1, ATF3/AP-1, CEBP/NFIL3) (Figure 5A). However, the relative order and frequencies of motifs recognized by SDTFs were different from those observed in the total set of LPS-induced enhancers. In particular, an IFN-sensitive response element (ISRE) recognized by IRF factors was the fourth most enriched motif in 3′SL-repressed enhancers and was present in nearly 22% of the targets. In contrast, the corresponding motif in the total set of LPS-induced enhancers ranked 5th and was present in only 5% of the targets (Figure 5A and Supplemental Figure 5A). Conversely, motifs recognized by NF-κB were ranked fourth and were present in 6% of the total set of LPS-induced enhancers but ranked tenth and were present in less than 1% of 3′SL-repressed enhancers (Figure 5A and Supplemental Figure 5A). Similar results with a greater representation of NF-κB motifs were obtained when evaluating the H3K27ac ChIP-Seq data just using the ATAC-Seq–defined enhancers activated by LPS (Supplemental Figure 5B).

These observations support the concept that both the 3′SL-sensitive and -insensitive enhancers are selected by a common set of macrophage lineage–determining factors (37) but that the motifs for SDTFs that act upon these enhancers differ. To further explore the role of NF-κB, we overlapped the 3′SL-sensitive enhancers with the DNA binding pattern of the p65 subunit of NF-κB following LPS stimulation. This analysis indicated that, despite consensus κB motifs being present at less than 1% of the 3′SL repressed enhancers, ~44% were occupied by p65 (Figure 5B). This result suggests that p65 binding at these locations is mediated by weak motifs and/or by indirect mechanisms. Functionality of p65 at these locations is in line with the upstream signal transduction regulatory analysis of the RNA-Seq data, indicating that the pattern of inhibition mediated by 3′SL is similar to that resulting from inhibition of inducers of NF-κB responses, such as IL-1β, IL-6, TNF, and the NF-κB activator MyD88 in LPS-stimulated BMDMs (Figure 5C). Furthermore, LPS-dependent induction of both H3K27ac and p300 recruitment was substantially reduced at local NF-κB/p65–bound enhancers (Figure 5, D and E). 3′SL alone did not regulate H3K27ac levels or p300 recruitment at these NF-κB/p65 enhancers in the absence of LPS stimulation (Figure 5, D and E). Examples of the relationships of p65 binding to 3′SL-repressed enhancers are exemplified at the *Il1a*, *Il6*, *Edn1,* and *Saa3* genes in Figure 5F and Supplemental Figure 5C. In contrast, *Cxcl10*, *Mx1*, *Socs3*, and *Ifnb* represent LPS-induced genes that are not repressed by 3′SL and show no changes in H3K27ac (Figure 5G and Supplemental Figure 5D).

The finding that a subset of 3′SL-repressed enhancers are occupied by p65 but lack consensus kB motifs led us to modify the motif enrichment analysis such that all LPS-induced enhancers were used as the background, rather than a GC-matched random genomic background. This approach eliminates motifs that are common to 3′SL-sensitive and 3′SL-insensitive enhancers, such as PU.1 and AP-1, and results in identification of motifs that are enriched in one subset but not the other. This analysis resulted in the identification of several motifs that occur at frequencies that are consistent with functional importance, including an ISRE (18%) recognized by IRFs, a motif recognized by the chromatin conformation modulator CTCF (8%), and a motif recognized by established repressor B cell lymphoma 6 (BCL6) (4%) (Figure 5H). Notably, a consensus NF-κB is not significantly enriched, supporting the concept that localization of p65 to a subset of 3′SL-sensitive enhancers is mediated by indirect interactions. Collectively, these findings provide evidence that 3′SL represses a select subset of LPS-induced genes by suppressing the activity of enhancers that lack consensus NF-κB motifs and are instead regulated by distinct combinations of SDTFs.

### Systemic 3′SL administration in Ldlr^–/–^ mice attenuates atherogenesis.

**Figure 6:**
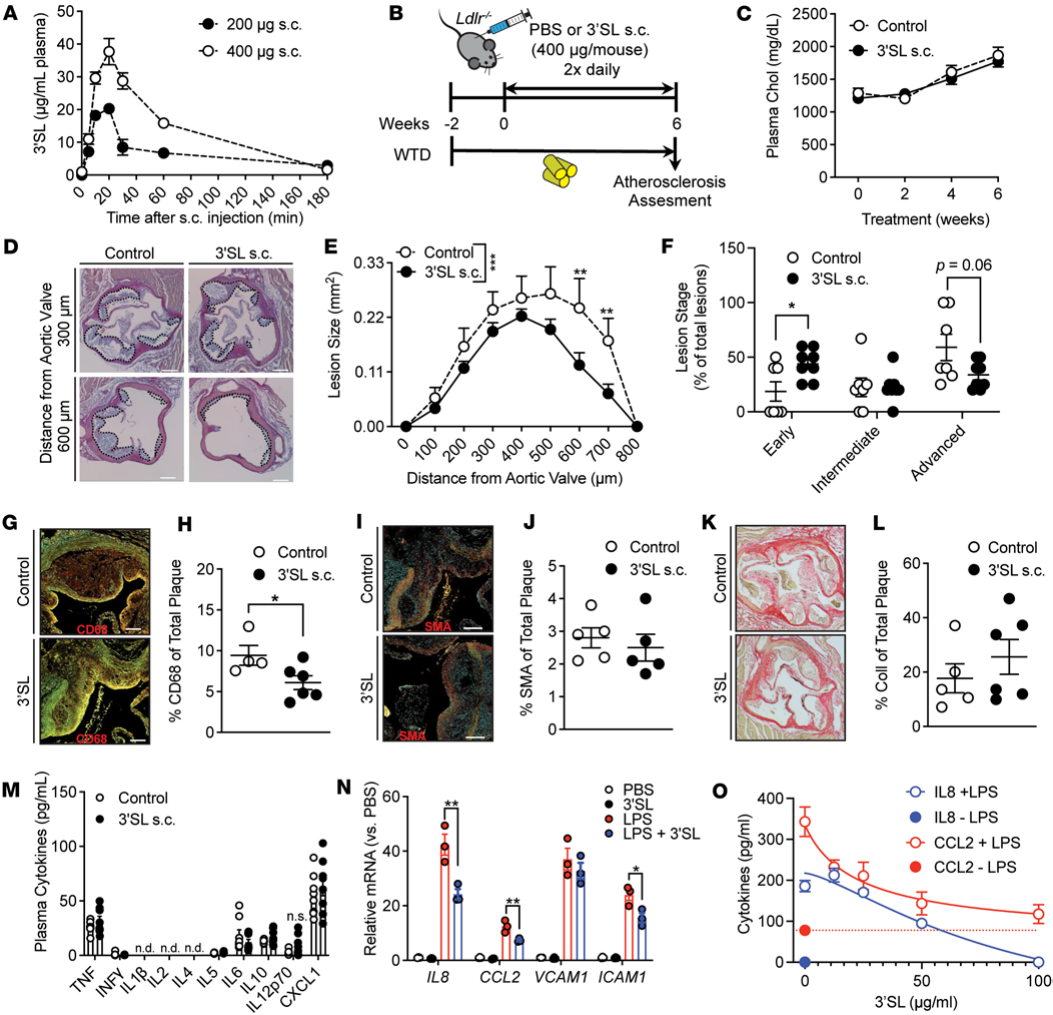
Six-week s.c. treatment with 3′SL leads to reduced atherosclerotic development without negative side effects associated with body weight, plasma lipid parameters, and blood glucose. (**A**) Pharmacokinetics of 3′SL after s.c. injections (*n* = 3 per group/time point). (**B**) Treatment regimen. Male *Ldlr^–/–^* were put on a Western-type diet (WTD) for 8 weeks. After 2 weeks, mice were treated twice daily with s.c. injections of 400 μg 3′SL in PBS per mouse for 6 weeks. PBS injections served as a control. (**C**) Biweekly plasma cholesterol levels in 3′SL-treated and control *Ldlr^–/–^* mice (*n* = 14–15). (**D** and **E**) Representative H&E staining (**D**) and quantification of atherosclerotic lesion size (**E**) in the aortic sinus. Scale bar: 100 μm (*n* = 7–8). (**F**) Atherosclerotic lesion staging analysis (*n* = 7–8). (**G** and **H**) Atherosclerotic lesions stained with CD68 for macrophages (**G**) and quantification (**H**) of the positive stained area (*n* = 4–6). (**I** and **J**) Smooth muscle actin (SMA) staining (**I**) and quantification (**J**) of the positive stained area (*n* = 5–6). (**K** and **L**) Picrosirius red staining for collagen (**K**) and quantification (**L**) of the positive stained area (*n* = 5). (**M**) Cytokine concentrations in the plasma after 6 weeks of treatment (*n* = 6). (**N**) Relative expression of inflammatory marker mRNA levels in human umbilical vein endothelial cells (HUVECs) treated with PBS or LPS (10 ng/mL) ± 3′SL (100 μg/mL) (*n* = 3). (**O**) Cytokine secretion of CCL2 and IL-8 after 24-hour stimulation with LPS in the presence or absence of 3′SL at the indicated concentrations (*n* = 3). Two-way ANOVA with Fisher’s LSD test and, in **H**, **J**, and **L**, we used an unpaired 2-tailed Student’s *t* test. *P < 0.05; **P < 0.01; ***P < 0.001. Data represent mean ± SEM.

Given the importance of chronic inflammation and inflammation resolution in atherosclerosis, we asked if 3′SL administration could be used therapeutically. We administered 3′SL via s.c. injections of Western-type diet (WTD: 42 % kcal from fat, 0.2 % total cholesterol) fed *Ldlr^–/–^* mice, as oral administration of 3′SL alters the gut microbiome and could confound interpretation of results (14, 15). Based on the blood volume of an average mouse (77–80 mL/kg) we evaluated the pharmacodynamics of a 400 μg and 200 μg s.c. 3′SL injection in 200 μL PBS (Figure 6A). Pharmacokinetic analysis showed that 3′SL appeared in the circulation within 5 minutes after s.c. injection. The concentration peaked at 20 minutes (20 and 35 µg/mL plasma, respectively) and returned to the baseline after 180 minutes (Figure 6A). Based on the rapid clearance of 3′SL, we injected 400 μg 3′SL or equal volume of PBS control s.c. twice daily as this concentration was within our effective IC_50_ dose tested in vitro (Figure 1G). Male *Ldlr^–/–^* mice were given WTD 2 weeks prior to initiation of 3′SL treatment to induce hypercholesterolemia, and this continued for the remainder of the experiment. Two weeks after initiation of the WTD, *Ldlr^–/–^* mice were injected with 3′SL (400 μg in 200 μL PBS) or PBS (200 μL) twice daily for 6 weeks (Figure 6B). 3′SL therapy did not alter food intake, body weight gain, or organ weights of adipose tissues or liver at the time of harvest compared with PBS control–treated *Ldlr^–/–^* mice (Supplemental Figure 6, A–C). Plasma lipid levels and lipoprotein profiles were not altered by 3′SL intervention (Figure 6, C and D, and Supplemental Figure 6, D and E). Fasting blood glucose or hepatic lipid content did not change (Supplemental Figure 6, F and G). This establishes that 3′SL administration did not alter the atherosclerotic lipoprotein drivers in *Ldlr^–/–^* mice.

We next examined the effect of 3′SL on atherogenesis. Atherosclerosis lesion area was assessed via en face aorta analysis (Supplemental Figure 6H) and lesion volume via serial aortic root analysis (Figure 6, E and F, and Supplemental Figure 6I). The short 8-week WTD feeding regimen resulted in very little en face plaque in the aorta (>2% of aortic surface area), which was not different between treatment groups (Supplemental Figure 6H). In contrast, analysis in the aortic root, where lesions are more advanced in mice, revealed that 3′SL treatment reduced lesion volume by 30% (1.4 ± 0.2 mm^3^ versus 0.96 ± 0.09 mm^3^; *P* = 0.002) (Figure 6, E and F). These data imply that therapeutic 3′SL administration reduces development of WTD-induced atherosclerosis in *Ldlr^–/–^* mice independent of changes in plasma lipoprotein levels.

### Intervention with 3′SL reduces macrophage lesion content and improves plaque stability markers.

We also evaluated the effect of 3′SL treatment on atherosclerotic plaque cell and extracellular matrix composition. We initially quantified lesion macrophage content via CD68 immunofluorescence staining. 3′SL therapy was associated with a significant 1.5-fold reduction in macrophage content in 3′SL mice when compared with equal-sized lesions from PBS-treated *Ldlr^–/–^* mice (6.1% of lesion area versus 9.4%; *P* < 0.05) (Figure 6, G and H). No differences in smooth muscle cell content but a slight trend toward increased collagen content in 3′SL-treated mice were noted (Figure 6, I–L). However, despite a reduction in macrophage content, visual inspection of van Gieson–stained cross-sections of lesions at the aortic root did not show differences in necrotic core content between the treatment groups (Supplemental Figure 6I), nor did we observe alterations in systemic plasma cytokine concentrations after 6 weeks of treatment between the treatment groups (Figure 6M). Plasma monocyte, neutrophil, and lymphocyte counts were also unchanged (Supplemental Figure 6, J and K). Although we previously showed that 3′SL does not directly interfere with monocyte’s rolling and adhesion to activated endothelium (38), we also evaluated if 3′SL promotes comparable antiinflammatory properties on activated endothelium. Stimulation of human umbilical vascular endothelium cells (HUVEC) with (10 ng/mL) LPS-induced mRNA levels of known factors that promote monocyte recruitment and invasion such as *Il8*, vascular adhesion molecule-1 (*VCAM1*), intercellular adhesion molecule-1 (*ICAM1*), and the monocyte chemoattractant protein C-C motif chemokine ligand 2 (*CCL2*) (Figure 6, N and O). Treatment of HUVEC with 3′SL alone did not affect the expression of these inflammatory genes. However, cotreatment of HUVEC with LPS and 3′SL (100 μg/mL) significantly reduced the expression of adhesion molecule *ICAM1*, chemoattractant proteins IL-8, and CCL2 by 1.5-fold (Figure 6, N and O), supporting the idea that 3′SL could inhibit trafficking of monocytes into an inflamed artery. Overall, the findings suggest that s.c. 3′SL treatment attenuates atherosclerosis development by reducing the number of macrophages in the lesions.

### Oral 3′SL administration attenuates atherogenesis in Ldlr^–/–^ mice.

**Figure 7:**
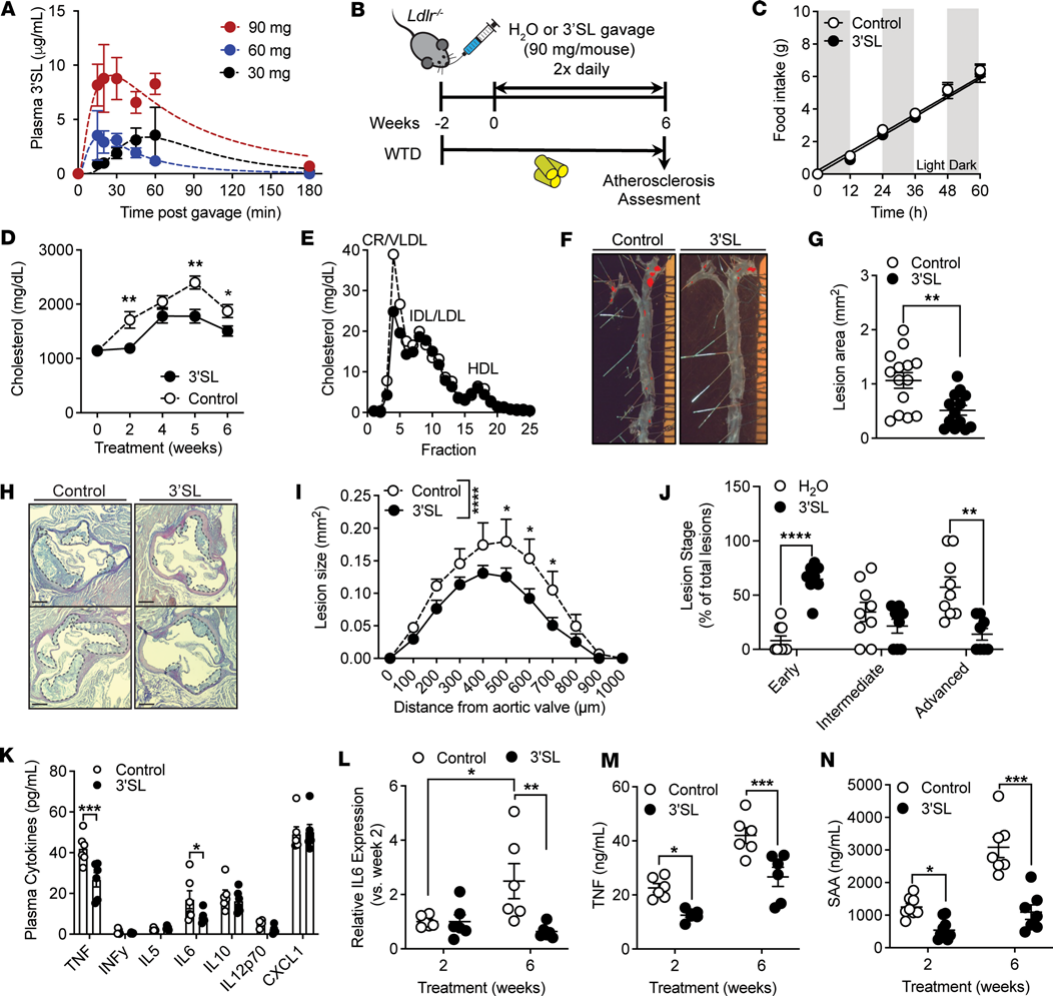
Six-week oral 3′SL treatment reduced atherosclerotic development and associated inflammation. (**A**) Pharmacokinetics of 3′SL after oral injections (*n* = 3 per group/time point). (**B**) Treatment regimen. Male *Ldlr^–/–^* were put on a Western-type diet (WTD) for 8 weeks. After 2 weeks, mice were treated twice daily with oral gavage of 90 mg 3′SL in water per mouse for 6 weeks. Water gavage served as a control. (**C**) Food intake of *Ldlr^–/–^* mice on a WTD measured 3 weeks into the treatment. (**D**) Biweekly plasma cholesterol levels in 3′SL-treated and control *Ldlr^–/–^* mice (*n* = 14–15). (**E**) FPLC cholesterol lipoprotein profiles after 6 weeks of treatment (2 pooled samples per group of 7–8). (**F** and **G**) Representative Oil Red O staining (**F**) and en face quantification of atherosclerotic lesion size (**G**) in the aorta (*n* = 14–15). (**H** and **I**) Representative H&E staining (**H**) and quantification of atherosclerotic lesion size (**I**) in the aortic sinus. Scale bar: 100 μm (*n* = 14–15). (**J**) Atherosclerotic lesion staging analysis (*n* = 8–9). (**K**) Cytokine concentrations in the plasma after 6 weeks treatment (*n* = 6). (**L**) Relative plasma IL-6 levels after 2 and 6 weeks of treatment (*n* = 6). (**M**) Plasma TNF levels after 2 and 6 weeks of treatment (*n* = 7–8). (**N**) Plasma SAA levels after 2 and 6 weeks of treatment (*n* = 6). Two-way ANOVA with Fisher’s LSD test and, in **G**, unpaired 2-tailed Student’s *t* test. *P < 0.05; **P < 0.01; ***P < 0.001; ****P < 0.0001. Data represent mean ± SEM.

We next investigated if oral 3′SL administration reduced atherosclerosis development as it would be a more clinically relevant therapeutic option. We determined absorption of orally administered 3′SL using different doses (30, 60, and 90 mg per mouse) into recipient WT mice (Figure 7A). Oral 3′SL administered via a single bolus in 200 μL administered via oral gavage appeared in the blood stream within 5 minutes after the bolus, reaching a peak 20 min lasting up to 60 minutes after gavage and returned to baseline after 180 minutes. Oral delivery of 90 mg 3′SL resulted in peak concentrations of 9–12 μg/mL for at least 60 minutes after administration (Figure 7A), which is in range of our effective IC_50_ dose tested in vitro (Figure 1G). No adverse effects were seen with all the tested concentrations, nor did they result in development of undesirable gastrointestinal side effects. Therefore, we fed *Ldlr^–/–^* mice with a WTD 2 weeks prior to initiating a twice-daily oral gavage of 90 mg/mouse 3′SL in 200 μL water, or 200 μL water via oral gavage as a control, for 6 weeks (Figure 7B). The twice-daily administration was chosen to maximize the systemic 3′SL exposure akin to the s.c. treatment protocol. This concentration and treatment regimen was well tolerated and did not result in development of loose stools (or diarrhea) or any undesirable gastrointestinal side effects. 3′SL therapy did not alter food intake (Figure 7C), body weight gain, or organ weights of adipose tissues or liver at the time of harvest compared with control-treated *Ldlr^–/–^* mice (Supplemental Figure 7, A and B). Plasma lipid levels and lipoprotein profile analysis showed a drop in plasma very low–density lipoprotein–associated (VLDL-associated) triglyceride and cholesterol levels in 3′SL-treated mice (Figure 7, D and E, and Supplemental Figure 7, C and D). However, no differences in the atherogenic LDL-cholesterol levels were observed between the treatment groups (Figure 7E). Similarly, fasting blood glucose and hepatic lipid content were unaltered (Supplemental Figure 7, E–G).

We next examined the effect of oral 3′SL administration on atherogenesis. Atherosclerosis lesion area and volume were assessed via en face aorta and serial aortic root analysis, respectively (Figure 7, F–I). Compared with control-treated mice, the 6-week 3′SL administration resulted in a significant 51% reduction in en face atherosclerotic lesion area staining in the aorta (1.064 ± 0.1436 mm^2^ versus 0.5129 ± 0.0898 mm^2^; *P* < 0.01) (Figure 7, F and G). In accordance, aortic root plaque analysis revealed that 3′SL treatment reduced lesion volume by 40% (1.0 ± 0.2 mm^3^ versus 0.6 ± 0.07 mm^3^; *P* < 0.0001) (Figure 7, H and I). Lesion analysis revealed no significant differences in necrotic core, collagen, macrophage, and smooth muscle cell content between the treatment groups (Supplemental Figure 7, H–K). These data imply that oral 3′SL administration significantly attenuated development of WTD-induced atherosclerosis in *Ldlr^–/–^* mice.

### Oral 3′SL administration in Ldlr^–/–^ mice reduces atherosclerosis-associated inflammation.

In addition to lesion development, we were interested in whether atherosclerosis-associated inflammation was affected by oral 3′SL administration. Plasma cytokine analysis revealed that, after 6 weeks of 3′SL treatment, plasma TNF and IL-6 levels were significantly reduced by 1.6- and 2-fold, respectively, compared with levels in control-treated *Ldlr^–/–^* mice (Figure 7J). Two weeks into the treatment, plasma IL-6 levels did not differ between 3′SL- and control-treated *Ldlr^–/–^* mice (Figure 7K). After 6 weeks IL-6 levels significantly increased by 2.5-fold in control treated *Ldlr^–/–^* mice but showed a 1.5-fold decrease in 3′SL-treated *Ldlr^–/–^* mice compared with plasma levels at 2 weeks (Figure 7L). Oral 3′SL administration reduced plasma TNF by 1.8-fold at 2 weeks into the treatment and remained significantly lower throughout the treatment (Figure 7M). In addition, we also measured SAA in both 3′SL- and control-treated *Ldlr^–/–^* mice (Figure 7N). SAA is an acute phase protein, and its plasma levels correlate with atherosclerotic development in mice and humans (39). In mice, SAA serves as a proxy for the human atherogenic hsCRP, as CRP in mice is only a modest acute phase protein (40). Oral 3′SL treatment was associated with significantly reduced plasma SAA levels after 2 and 6 weeks of treatment, by 2.3- and 2.8-fold respectively, compared with control-treated *Ldlr^–/–^* mice (Figure 7M). In conclusion, the findings suggest that oral 3′SL treatment reduces atherosclerosis development and attenuates atherosclerosis-associated inflammation in WTD fed *Ldlr^–/–^* mice.

### 3′SL administration in Ldlr^–/–^ mice reduces inflammatory gene expression in myeloid cells in atherosclerotic lesions.

**Figure 8:**
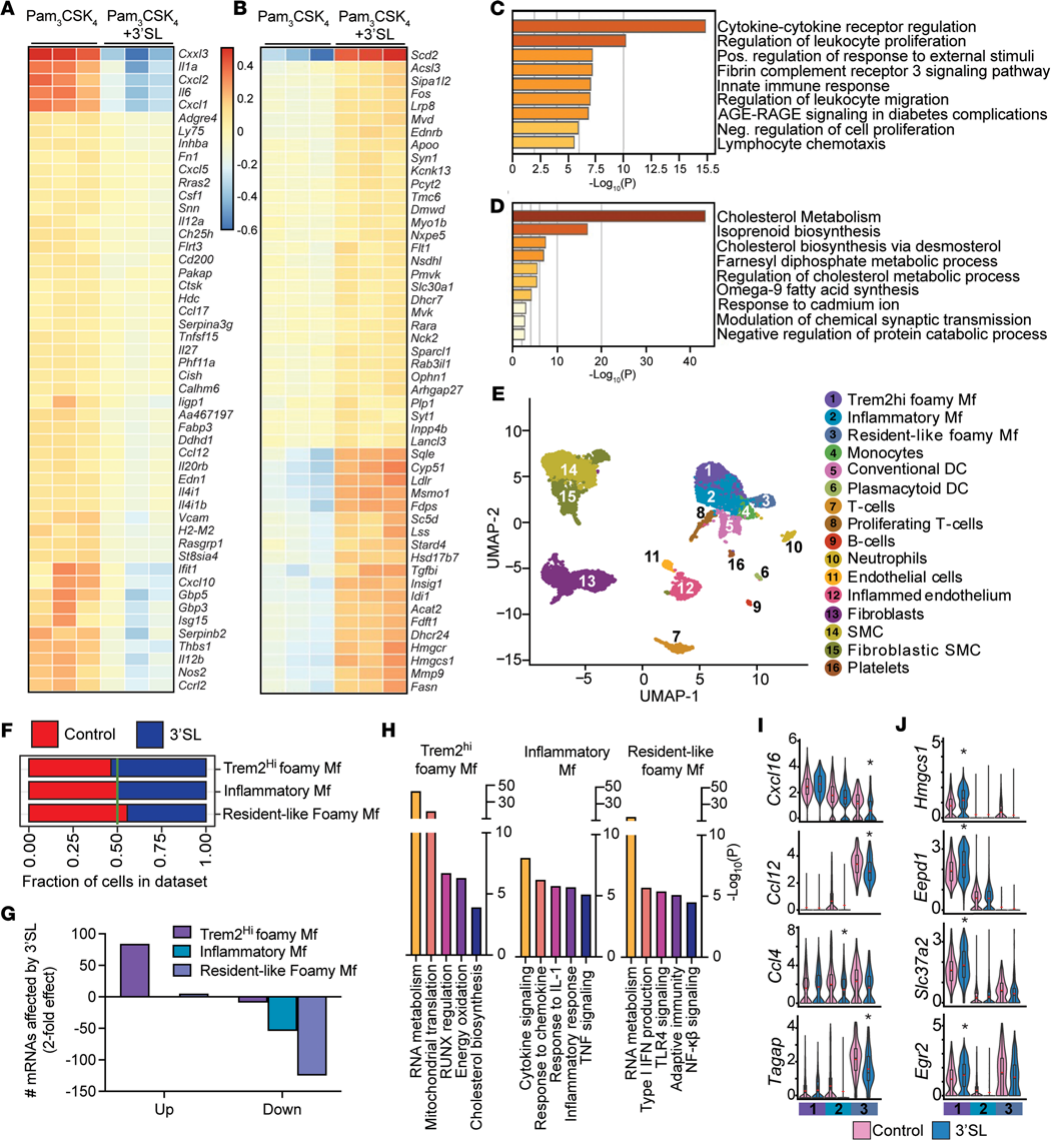
3′SL downregulates inflammatory pathways and upregulates cholesterol biosynthesis in atherosclerotic lesion macrophages. (**A** and **B**) Heatmaps representing *Z*-normalized row mRNA levels of each gene for RNA-Seq from independent biological duplicates showing the 50 most upregulated (right) or downregulated (left) genes in BMDMs at 6 hours after Pam3CSK4 ± 3′SL stimulation. (**C** and **D**) Enriched pathways identified by metascape analysis software. All significantly, differentially expressed mRNA levels of genes in the Pam3CSK4 versus Pam3CSK4 + 3′SL RNA-Seq datasets were used for the analysis with IPA (cut-off > 1.5-fold) with *Z* score ≥ ±2. (**E**) Annotated UMAP visualizations of the identified clusters of snRNA-Seq data from cells of the atherosclerotic aortic arch of water treated or 3′SL-treated male mice. *Ldlr^–/–^* male mice were fed a Western-type diet (WTD) for 10 weeks. After 2 weeks, mice were treated twice daily with oral gavage of 90 mg 3′SL in water per mouse for 6 weeks. Water gavage served as a control. (**F**) Relative distribution of atherosclerotic lesion macrophage subsets in water versus 3′SL-treated mice. (**G**) The number of significantly differentially expressed mRNA gene transcripts in macrophage subsets and their directionality upon 3′SL administration (upregulation is > 2-fold; downregulation is <-2-fold; compared with control treatment). (**H**) Enriched pathways identified by metascape analysis software of differentially expressed mRNA in macrophage subsets upon 3′SL administration (> 1.75-fold or < –1.75 change; compared with control treatment). (**I** and **J**) The expression of numerous genes from snRNA-Seq was significantly increased (**I**) or decreased (**J**) by 3′SL treatment in plaque macrophage subsets compared with WT. Unpaired 2-tailed Student’s *t* test. *P < 0.05 versus control. Data represent mean ± SEM.

We next investigated if inflammation reduction was also promoted by 3′SL at the atherosclerotic lesion level. Typical activators of inflammation in lesion macrophages are not driven by LPS or TLR4. In atherosclerotic lesions, activation of TLR2 by modified lipid species (oxLDL) occurs. Hence, we evaluated if 3′SL treatment induces a similar pattern of modulation of inflammatory genes and stimulation of LXR/SREBP responsive genes upon TLR2 activation, as observed with 3′SL and LPS stimulation (Figure 3). Costimulation of BMDMs with the TLR2 agonist PAM3CSK4 (100 μg/mL) and with 3′SL (100 μg/mL) for 6 hours did repress activation of inflammatory genes (Figure 8, A and C). The cotreatment also significantly promoted expression of genes predominantly involved in cholesterol biosynthesis and fatty acid metabolism (Figure 8, B and D). This is independent of macrophage foam cell formation, which was not affected by 3′SL (Supplemental Figure 8, A–C). We next performed single-nuclei RNA-Seq (snRNA-Seq) on atherosclerotic lesions to identify if similar antiinflammatory responses could be observed in lesion-associated innate immune cell populations. To isolate adequate lesions quantities, especially in the 3′SL treatment group, we fed *Ldlr^–/–^* mice with a WTD 2 weeks prior to initiating a twice-daily oral gavage of 90 mg/mouse 3′SL in 200 μL water, or 200 μL water as a control, for 10 weeks, instead of 6 weeks (Figure 7B). We isolated all atherosclerotic lesions from the ascending aorta and the aortic arch and subjected them to single-nuclei isolation to allow an unbiased snRNA-Seq of all lesion-associated cell populations (Figure 8E). Population size differences of the macrophage subsets were observed between control and 3′SL treatment groups (Supplemental Figure 8D). The 3′SL treatment was associated with a reduction in the proliferative resident foam cell macrophages and enrichment of the antiinflammatory Trem2^hi^ macrophage foam cells that typically express mRNAs involved in cholesterol biosynthesis and fatty acid metabolism (Figure 8, F and G, and Supplemental Figure 8D). TREM2 is expressed on antiinflammatory macrophages and has been shown to limit macrophage activation (41). Pathway analysis of the gene mRNA transcript population that were significantly (*P* < 0.05) altered by 3′SL treatment revealed enrichment for genes involved in reducing inflammation (M2-like) and TLR2 inflammation as well as a response to RXR signaling (Supplemental Figure 8E). Interestingly, many of the upregulated genes were associated with the Trem2^hi^ macrophage population, while downregulation of mRNA transcripts was confined to inflammatory macrophages and resident-like foam-like macrophages (Figure 8G). Pathway analysis of the affected genes in the different macrophage populations revealed that most of the downregulated mRNAs converged on inflammation and cytokine signaling (Figure 8H). The upregulated mRNA transcripts in the antiinflammatory Trem2^hi^ macrophage foam cells belonged to genes involved in mRNA and mitochondrial activity as well as oxidative phosphorylation, RUNX activity, and cholesterol biosynthesis (Figure 8H). Among the downregulated mRNAs were proinflammatory genes such as *Ccl4*, *Ccl12*, *Ccl16*, *Isg15*, *Il6,* and *Tagap* in the inflammatory macrophage population (Figure 8I). mRNA transcripts that were upregulated in the 3′SL treatment group in the Trem2^hi^ macrophage lesion population comprised LXR responsive genes such as *Hmgcs1* and *Eepd1* as well as antiinflammatory macrophage markers *Egr2* and *Slc37a2* (Figure 8J). In contrast, fewer genes in the endothelial population were altered and not specifically associated with reduction or alteration in the inflammatory status (Supplemental Figure 8, F and G). To conclude, in the inflammatory setting of atherosclerotic disease, 3′SL treatment resulted in a less inflammatory phenotype of plaque macrophages.

## Discussion

This study describes the effect of 3′SL on reducing low-grade chronic macrophage inflammation in the context of CVD. Our data support that the antiinflammatory properties of 3′SL are mediated via reduction of inflammatory gene expression but equally by accelerating expression of genes in macrophages under control of LXR and SREBP that are important in the initiation of the inflammation resolution phase (33). Based on the data, 3′SL treatment of macrophages appears to exert an atheroprotective role, by attenuating inflammation and reducing the expression of chemoattractants and cytokines that exponentially accelerate atherosclerosis development driven by hypercholesterolemia. Overall, our results support a concept that 3′SL can be used orally as a therapeutic in the context of adult diseases due to inhibitory effects on low-grade chronic inflammation and reduction of atherosclerotic lipoproteins.

Several reports have described induction of TLR4 signaling by specific glycan structures, including HMOs (42). The neutral HMOs LNFP II and LNnT affect peritoneal suppressor macrophages (43), while other studies show that acidic HMOs have various immunomodulatory properties (38, 44). In contrast, our study highlights the antiinflammatory effects of 3′SL in LPS-stimulated macrophages. Our results suggest that 3′SL, and to a lesser extent 6′SL, are antiinflammatory, yet the structurally related DSLNT is not. Our results are in line with other reports attributing antiinflammatory effects to 3′SL. Kang and colleagues investigated the role of 3′SL in inflammation connected to rheumatoid arthritis and observed that cotreatment with IL-1β and 3′SL in THP-1 cells also reduced IL-1β, IL-6, and TNF mRNA and protein expression (45). Also, 3′SL reduced cytokine production in other inflammation-relevant cell types. Kang and colleagues also attributed antiinflammatory effects to 3′SL in skin inflammation (46). Again, similar expression patterns can be observed, suggesting that the antiinflammatory effect of 3′SL persist across different inflammatory disease models and cell types. Also, it is important to note that we carefully removed LPS and other contaminations from 3′SL. Depending on the source and production method, 3′SL can contain a substantial amount of contaminants that affect the immunomodulatory effects as reported before (42, 47).

Both 3′SL and 6′SL are structurally almost identical, with the only difference being the sialic acid moiety linkage to galactose which is an α2,3 and α2,6 linkage, respectively. Acidic HMOs such as 3′SL, 6′SL, and DSLNT share similar linkages; however, DSLNT is sialylated by an α2,3 linkage to galactose and an α2,6 linkage to the internal *N*-acetyl glucosamine. Since each sialic acid contributes 1 negative charge of the HMO, the additional charge on DSLNT may interfere with its binding to a putative macrophage receptor. In addition, the molecular weight of DSLNT is nearly twice that of 3′SL or 6′SL (1,290.14 g/mol versus 633.55 g/mol). The difference in size and charge of the structure may hinder the steric interactions of DSLNT and the macrophage receptor. Using genetic approaches, we also excluded that 3′SL antiinflammatory properties are the result of simple engagement of the sialic acid, with most the abundant antiinflammatory Siglecs expressed on macrophages (23). We cannot exclude that the combined inactivation of all antiinflammatory Siglec receptors is essential to mitigate the antiinflammatory effect. The studies highlight that 3′SL and 6′SL have structure-specific antiinflammatory properties that are poorly understood. However, simply carrying a sialic acid moiety on the HMO lactose backbone or complex HMO is not sufficient. We did not test if other 3′sialylated (non-HMO) glycans are equally effective, but given the HMO’s structural specificity and the lack of DSLNT’s effect, we predict that 3′SL has optimal structural components to mediate the antiinflammatory effects. Previous studies identified that free sialic acid administration promotes inflammation in macrophages (48) and endothelial cells (49). In our previous work, oral sialic acid (Neu5Ac) administration to *Ldlr^–/–^* mice did not affect atherogenesis, inflammation, and lipid profiles, supporting the observation that sialic acid by itself is not sufficient to observe our antiinflammatory effects (50). It is unclear how HMOs like 3′SL enter the bloodstream intact. 3′SL might be absorbed through receptor-mediated uptake, transcytosis, or paracellular transport. It could circulate freely or be paired with carrier proteins or other molecules for stability. Investigating these mechanisms could shed light on 3′SL’s role in the body and its therapeutic potential. Combining 3′SL with 6′SL might also reveal synergistic effects, offering insights into immune response modulation.

Previous reports have suggested 3′SL inhibits NF-κB/p65 signaling in unstimulated Caco-2 cells, a cell model for enterocytes (51). At our effective dose, we did not observe any effect of 3′SL administration on NF-κB/p65 signaling. Instead, using an unbiased transcriptomic and epigenetic approach, we identified that the antiinflammatory effect of 3′SL on TLR4-activated macrophages is surprisingly focused on a selected group of affected genes and enhancers. In contrast, over 5,000 genes were differentially expressed in BMDMs in response to just TLR4 activation. The observation supports the idea that 3′SL does not globally inhibit TLR4 activation and NF-κB/p65 signaling. This concept is also corroborated by the observation that 3′SL attenuates TLR2-mediated macrophage activation, a well-established atherogenic pathway. Hence, our data also exclude that the antiinflammatory effect of 3′SL is a consequence of altering signaling events mediated by CD14 or myeloid differentiation factor 2 (MD2) independently (52). We so far have not identified the receptor responsible for the biological activity, and studies are underway to identify the relevant receptor pathway responsible for 3′SL’s transcriptional effects.

Unexpectedly, we found that 3′SL was able to induce expression of a set of genes that is regulated by LXR and SREBP1, master regulators of cholesterol and fatty acid biosynthesis (35, 36, 53). The modulation of lipid metabolism via these transcription factors is important for short-term immune cell activation as well as long-term trained innate immunity (33, 54–57). However, loss-of-function experiments indicated that LXRs and SREBPs were not required for the antiinflammatory effects of 3′SL on LPS-treated macrophages. Further studies will be required to determine the molecular mechanisms by which 3′SL activates LXR and SREBP and the functional consequences of these effects. Studies of the effects of 3′SL on the epigenetic response to LPS revealed inhibition of H3K27ac and recruitment of the p300 HAT at a select set of enhancer elements associated with 3′SL-suppressed genes. These results imply a transcriptional mechanism by which 3′SL inhibits LPS-induced gene expression that involves direct or indirect effects on sequence-specific transcription factors and/or their coactivators. In-depth de novo motif analysis of 3′SL-repressed enhancers indicated that they were qualitatively different from LPS-induced enhancers that were not subject to 3′SL repression. In addition to lack of consensus κB motifs, 3′SL-sensitive enhancers were distinguished by the presence of several other transcription factor–binding motifs. The most significantly enriched motif corresponds to a binding site for CTCF. CTCF is a highly conserved factor regulating gene transcription by spatially orienting *cis*-acting regulatory elements within topologically associating domains (58). The strong enrichment of 3′SL-repressed genes for the CTCF motif raises the intriguing possibility that 3′SL alters LPS responses at specific genes by influencing CTCF-dependent interactions between enhancers and promoters. Several additional motifs were preferentially enriched in 3′SL-sensitive enhancers, such as BCL6 and RBPJ, and canonically serve as transcriptional repressors (59). RBPJ is a Notch signaling modulator and a regulator of proinflammatory macrophages polarization via activation of IRF8 (60). The latter might explain why we also see enrichment for the IRF motif, despite that fact that most type I IFN responsive genes are unaffected by 3′SL coincubation (Supplemental Figure 5B). BCL6 encodes a BTB/POZ-zinc finger transcriptional repressor critical for the development and inflammatory potential of immune cells, including attenuation of NF-κB/p65 signaling in macrophages (59, 61). BCL6 and the NF-κB cistromes intersect, within nucleosomal distance, at nearly half of BCL6-binding sites in stimulated macrophages to promote opposing epigenetic modifications of the local chromatin (62). Recent studies in macrophages suggest that BCL-6 interacts with IκBζ and interferes its binding to the *IL6* promoter in macrophages (63). Further studies are needed to evaluate if 3′SL activates BCL6 recruitment directly or indirectly by activation of canonical ligand signaling pathways such as IL-4 and IL-21.

Importantly, we show that the antiinflammatory effects of 3′SL in vitro translated in attenuated atherosclerosis lesion development in vivo. Antiinflammatory effects of 3′SL in vivo were previously shown in mouse models of rheumatoid arthritis and dermatitis (45, 46). However, 3′SL was administered orally; thus, it cannot be excluded that the antiinflammatory phenotypes are due to likely drastic changes in the gut microbiome (14, 46). We intentionally sought to circumvent interactions of 3′SL with the microbiome using s.c. administration, without any negative side effects, as expected (64–66). The 3′SL in *Ldlr^–/–^* mice resulted in a reduction in atherosclerotic lesion size and macrophage content, in line with in vitro reduced expression and secretion of proinflammatory cytokines and chemoattractants. In addition, we also observed that 3′SL administration reduced expression of endothelial chemokine and adhesion molecules that promote monocyte infiltration in cultured endothelial cells. It is very likely that both processes contribute to the attenuated atherogenesis and reduced plaque macrophage content. Based on previous reports, we know that 3′SL does not directly compete with binding of monocytes to adhesion factors expressed by endothelium (38). Hence, it will be important to probe in the future the effect of 3′SL administration on established atherosclerotic lesion regression and plaque rupture, which lead to relevant clinical events.

Finally, we also assessed the effect of oral 3′SL treatment, which is clinically more relevant as a therapeutic strategy, to determine whether it reduced atherosclerosis even more strongly compared with a s.c. 3′SL intervention. A key difference between the 2 administration routes were triglyceride-rich lipoprotein (TRL) levels, which were reduced by oral but not by s.c. 3′SL treatment. However, most TRL-lowering strategies fail to see an effect on atherosclerosis development in murine hypercholesterolemia models, unless they can also lower LDL-cholesterol levels (67). Thus, it remains to be determined if this reduction contributes to the antiatherogenic effect and whether the TRL-lowering effect is a result of reduced lipid absorption and VLDL production or accelerated hepatic clearance. Such findings are likely not only important under high-fat feeding diets conditions in adults, but they could also be relevant for infant lipid metabolism, as infants consume a very high-fat meal on a frequent basis. 3′SL could be degraded or interact with endogenous 3′-neuraminidases, influencing signaling pathways. If degraded, 3′SL’s availability for receptor interaction could decrease, reducing its effectiveness. Alternatively, 3′SL might inhibit or compete with neuraminidases, altering the balance of sialylated substrates and affecting downstream signaling or receptor engagement. Understanding 3′SL’s stability and interactions with enzymes is key to clarifying its mechanisms and effects.

We cannot rule out that the effects on systemic and lesion inflammation during oral gavage might stem from secondary effects on the liver or gut. The observation that SAA went down a great deal upon oral 3′SL treatment is strong evidence for a systemic decrease in inflammation as SAA is mainly made in the liver (40). This favorable antiatherogenic discrepancy between the 2 administration routes could be a result of alterations in the gastrointestinal microbiome or due to a first-pass effect of oral administered 3′SL via the liver, where the majority of the measured cytokines and TRLs are produced and cleared. Finally, the snRNA-Seq analysis of lesion macrophages in 3′S- treated *Ldlr^–/–^* mice mirrored our in vitro results. We observed a reduction of inflammatory genes in the inflammatory foam cell macrophages and an increase in LXR target genes and lipid biosynthetic genes in the Trem2^hi^ macrophage population (41). Despite finding no gross changes in macrophage lesion content, the analysis confirmed that 3′SL also at the lesion level reduces the inflammatory potential of lesion macrophages, the most abundant cell type in the plaques. Beyond effects on macrophages, we also observed significant alterations in the mRNA profiles of lesion plasmacytoid DC, proliferating T cell populations, and fibroblasts; however, no gross effects were seen in endothelium (Supplemental Figure 8, F–H). Many of these gene expression alterations are not related to atherosclerosis, and future studies need to evaluate how these changes influence atherogenesis and plaque evolution.

With 3′SL being a natural compound that received GRAS (Generally Recognized As Safe) status by the United States FDA, it could provide a viable alternative solution for treating chronic inflammation and atherosclerosis and CVD. Moreover, antiinflammatory therapeutics are favorable over agents that directly block cytokine release because they are less likely to compromise the patient’s immune system. Hence, our data warrant further analysis of 3′SL and its therapeutic potential in CVDs and other chronic inflammatory disorders.

## Methods

Supplemental Methods are available online with this article.

### Sex as a biological variable.

Our cohort is balanced for sex to ensure an equal number of male and female individuals were included in both arms of the study. However, for the atherosclerosis evaluation, we examined male mice because male animals exhibited less variability in phenotype.

### Statistics.

Statistical analyses were performed using Prism software (version 7, GraphPad Software). Data were analyzed by 2-tailed unpaired Student’s *t* test and presented as mean ± SEM. For experiments with more than 2 experimental groups, statistical significance was determined by ANOVA for multiple comparisons with Fisher’s LSD post hoc tests for multiple pair-wise comparisons. *P* < 0.05 was considered significant.

### Study approval.

All experimental procedures were approved by the UCSD IACUC.

### Data availability.

The raw data of snRNA-seq are publicly available at GSE167999, PRJNA996113 (https://www.ncbi.nlm.nih.gov). Supporting Data Values for all figures are provided within the supplement.

## Author contributions

LB and PLSMG conceived the project. ARP, NJS, CAA, KZ, HMH, NEL, JLW, RY, ARA, MD, CDP, KVG, LW, MPJDW, RME, CKG, LB, and PLSMG designed experiments. ARP, NJS, CAA, KVG, CDP, TGO, JKCC, BR, JL, AH, AWTC, JH, CYW, APC, UO, NEL, MPJDW, YW, AQ, MP, AWTC, CT, and LMB analyzed data and performed experiments. ARP, NJS, CAA, ARA, MD, RME, CKG, LB, and PLSMG interpreted data and wrote the manuscript, and the final versions were reviewed by all authors. ARP and NJS are co–first authors; ARP is listed first, as she initiated the work.

## Supplementary Material

Supplemental data

Unedited blot and gel images

Supporting data values

## Version 1 09/26/2024

In-Press Preview

## Version 2 11/08/2024

Electronic publication
