# Supplementary material for: The human milk oligosaccharide 3′sialyllactose reduces low-grade inflammation and atherosclerosis development in mice
Source: JCI Insight. 2024 Nov 8;9(21):e181329. doi: 10.1172/jci.insight.181329 (PMC11601559; doi:10.1172/jci.insight.181329)
Supplement: Supplemental data [file jciinsight-9-181329-s139.pdf]

SUPPLEMENTAL DATA TABLES AND FIGURES

The Human Milk Oligosaccharide 3'Sialyllactose Reduces Low-grade Inflammation and Atherosclerosis Development in Mice.

Ariane R. Pessentheiner[1,2*], Nathanael J. Spann[3,*], Chloe A. Autran[4], Tae Gyu Oh[5], Kaare V. Grunddal[1], Joanna K.C. Coker[1,6], Chelsea D. Painter[1], Bastian Ramms[1], Austin W.T. Chiang[4,6,7], Chen-Yi Wang[8,9], Jason Hsiao[1], Yiwen Wang[1], Anthony Quach[1], Laela M. Booshehri[4], Alexandra Hammond[4], Chiara Tognaccini[1], Joanna Latasiewicz[4], Lisa Willemsen[10], Karsten Zengler[6,11], Menno P.J. de Winther[10], Hal M. Hoffman[1,4,12], Martin Philpott[8], Adam P. Cribbs[8,9], Udo Oppermann[8,9], Nathan E. Lewis[5,6,7], Joseph L. Witztum[1], Ruth Yu[5], Annette Atkins[5], Michael Downes[5], Ron M. Evans[5], Christopher K. Glass[1,3], Lars Bode[4,13,14,#] and Philip L.S.M. Gordts[1,14,#]

[1]Department of Medicine, University of California San Diego (UCSD), La Jolla, CA, USA, [2]Institute of Molecular Biosciences, University of Graz, Austria, [3]Department of Cellular and Molecular Medicine, [4]Department of Pediatrics at UCSD, La Jolla, CA, USA, [5]Gene Expression Laboratory, Salk Institute for Biological Studies, La Jolla, CA, USA, [6]Department of Bioengineering at UCSD, La Jolla, CA, USA, [7]Novo Nordisk Foundation Center for Biosustainability, La Jolla, CA, USA, [8]Botnar Research Centre, Nuffield Department of Orthopedics, Rheumatology and Musculoskeletal Sciences, National Institute of Health Research Oxford Biomedical Research Unit (BRU), [9]Oxford Centre for Translational Myeloma Research University of Oxford, Oxford, UK, [10]Department of Medical Biochemistry, Experimental Vascular Biology, Amsterdam Cardiovascular Sciences, Amsterdam Infection and Immunity, Amsterdam UMC, University of Amsterdam, Amsterdam, the Netherlands, [11]Center for Microbiome Innovation, UCSD , La Jolla, CA, USA, [12]Rady Children's Hospital of San Diego, San Diego, CA, USA, [13]Larsson-Rosenquist

Foundation Mother-Milk-Infant Center of Research Excellence (MOMI CORE), and [14]Glycobiology Research and Training Center, UCSD, La Jolla, CA, USA.

***These authors contributed equally.**

[#]To whom correspondence should be addressed:

Philip L.S.M. Gordts, Department of Medicine, UCSD, La Jolla, CA 92093-0687, Ph: 858/246-0994, FAX: 858/534-5611, pgordts@health.ucsd.edu

Lars Bode, Department of Pediatrics, UCSD, La Jolla, CA 92093-0687, Ph: 858/246-1874, lbode@health.ucsd.edu

Supplemental Methods

Separation, purification and identification of HMO

Pooled HMO (pHMO) used for the experiments were isolated and prepared with a method described previously(1). Breast milk collected at different times post-partum, was donated by healthy women who gave birth at term. The milk from more than 50 women was pooled and centrifuged to remove the lipid layer. In addition, proteins were removed by precipitation from the aqueous phase with ice-cold ethanol. Ethanol was evaporated using a rotary evaporator and the remainder of the samples was lyophilized. Lactose and salts were removed by FPLC and samples were analyzed by HPLC, where HMO profiles containing less than 2% lactose were pooled and later used for experiments. Individual HMO used in experiments were either generously provided or purchased commercially (Supplemental Table 1). Endotoxin (LPS) was removed from all pHMO and individual HMOs used for in vitro experiments by Detoxi-Gel Endotoxin Removing columns (Pierce Thermo Scientific, Rockford, IL, USA) according to the manufacturer instructions. The whole procedure was performed under sterile conditions with the use of pyrogen-free UltraPure distilled water, ethanol 200 proof and sterile filter tips to avoid LPS contamination. After collection of the flow-through, containing HMO, the purified samples were frozen immediately and lyophilized until completely dry. To minimize the amount of LPS, the HMO samples were processed twice on the endotoxin removing columns.

Tissue culture

RAW 264.7 macrophages, HUVEC and THP-1 human monocytic cells were obtained from American Type Culture Collection (ATCC, Manassas, VA, USA). RAW264.7 cells were cultured in DMEM supplemented with 10% fetal bovine serum (FBS) and 0.1% Penicillin-Streptomycin (Pen-Strep) and THP-1 cells in RPMI supplemented with 10% FBS and 10 mM HEPES in a

humidified incubator with a 5% CO_2 at 37°C. Prior to experiments THP-1 cells were split into 12 well plates ($1x10^6$ cells per well) and differentiated into macrophages by adding 600 nM phorbol 12-myristate 13-acetate (PMA) for 48 h. Differentiated macrophages attached to plate surface were used for experiments. HUVECs were grown in HUVEC, grown in 100-mm diameter dishes in EGM-2 medium (Lonza). BMDMs were isolated from femur and tibia from both hind legs of C57BL/6J mice and cultured in DMEM/F12 (supplemented with 10% FBS, 0.1% Pen-Strep) and 20 ng/mL MCSF to stimulate macrophage differentiation at 37°C, with 5% CO_2. Medium with MCSF was refreshed every other day. On day 5 to 7 post isolation cells were seeded into 24-well plates (450,000 cells/well) and used on the following day for experiment. Bone marrow from *Siglece*$^{-/-}$ *and Siglec1*$^{-/-}$ mice were a kind gift from Victor Nizet (UC San Diego, La Jolla, CA). Peripheral blood monocytes (PBMCs) were isolated from small volumes of whole blood from healthy volunteers after informed consent, following a protocol for simple phlebotomy approved by the UCSD IRB/Human Research Protection Program, by Ficoll gradient and plated at 10^5 cells per well in media without serum in a 96-well plate. Monocytes were allowed to adhere for 3 hours at 37°C before the supernatant was replaced with media with 10% FCS and incubated overnight at 37°C. LPS stock medium (10 ng/mL LPS in DMEM) was prepared and used for all cells activated with LPS at the day of the experiment. As a control for non-activated cells, PBS was used. HMOs diluted in H_2O were added in the concentration needed. To avoid microbial contamination all prepared media were filter sterilized (0.22 μm filter units). When other stimuli were used to induce inflammatory responses, preparation of media and cell handling followed the procedure explained above.

Mice

Ldlr$^{-/-}$ and C57BL/6 mice were purchased from The Jackson Laboratory and bred in our animal facility. *Lxrα/β* knock-out mice (*Lxr*$^{-/-}$) were generated as described(2). All animals were housed

and bred in vivaria approved by the Association for Assessment and Accreditation of Laboratory Animal Care located in the School of Medicine, UCSD, following standards and procedures approved by the UCSD Institutional Animal Care and Use Committee. Mice were weaned at 4 weeks, maintained on a 12-hour light cycle, and fed ad libitum with water and standard rodent chow (PicoLab® Rodent Diet 20 5053) or a Western diet (TD.88137, Envigo Teklad) containing 42 % kcal from fat and 0.2% cholesterol. Littermate controlled mice received 3'sialyllactose citrate in PBS (400 µg/injection) or PBS via subcutaneous injections twice daily for 6 weeks. Littermate controlled mice received 3'sialyllactose citrate in water (90 mg/gavage) or water via oral gavage twice daily for 6 to 8 weeks. For all treatments, all mice within each cage received the same treatment to prevent potential ingestion of 3'SL excreted by the treatment group due to coprophagy. All experimental procedures were approved by the UCSD Institutional Animal Care and Use Committee.

Multiplex Enzyme-Linked Immunosorbent Assay

BMDM were plated in 12-well plates (450,000 cells/well) as described above. Cells were co-incubated for 24 hours with 10 ng/mL LPS or PBS and 100 µg/mL 3'SL or PBS and supernatants were collected for measuring cytokine production of BMDM using the MSD Multi-spot Assay system Proinflammatory Panel 1 (V-Plex, K15048D, Mesa Scale Diagnostics, Rockville, MD, USA) or IL6 or IL1β (R&D systems) according to the manufacturer's protocol. Cytokine levels in murine plasma were determined from EDTA-plasma from 4h fasted mice with the same V-Plex kit. For the human IL8 and CCL2 measurements we used standard ELISA from R&D systems.

Western Blot

Protein extracts were harvested with RIPA buffer (supplemented with proteinase inhibitor and PhosStop) from BMDMs seeded in 12-wells after indicated times of stimulation with PBS, LPS and 3'SL. The lysates were subjected to Western blot analyses (4-12% SDS-PAGE, 15 µg/lane) using phospho-p38 MAPK (Thr180/Tyr182, Cell signaling, #9211) and phospho-NFκB p65 (Cell signaling, 93H1); as loading control antibody beta-Actin (Sigma Aldrich, SAB3500350) was used.

Quantification of atherosclerotic lesions

Five hour fasted *Ldlr*$^{-/-}$ mice were perfused with 10 ml of PBS following cardiac puncture. The heart and ascending aorta down to the iliac bifurcation were removed and incubated in sucrose buffer or formalin, respectively. The hearts were processed and stained by the atherosclerosis core morphology group at UC San Diego as described(3). The isolated hearts were sectioned by cutting several 10 µm paraffin cross-sections starting with the first appearance of the first leaflet of the aortic valve until the last leaflet in 100 µm sections. Aortic root atherosclerosis was analyzed using modified Verhoeff-van Gieson elastic staining to enhance the contrast between the intima and surrounding tissues. At each 100 µm cross-section, the mean lesion size in each mouse was analyzed by computer-assisted morphometry (Image-Pro Plus 6.3, Media Cybernetics) by two investigators blinded to the study protocol. After removal of adventitial tissues, the aortas were incised longitudinally, pinned flat and stained for neutral lipids using Sudan IV. Images were acquired using a Leica MSV266 microscope with an attached Leica Ic80 HD camera.

Histology of atherosclerotic lesions

Necrotic core formation was analyzed on modified Verhoeff-van Gieson elastic stained aortic root cross-sectional lesions (300 µm and 600 µm from beginning of aortic root) using ImageJ analysis software (NIH). Necrotic core area was measured as a percentage of total plaque area. Collagen

content was quantified as percentage of total lesion area in aortic root cross-sectional lesions (300-400 μm from beginning of aortic root) stained with Picrosirius Red. Immunohistochemical analysis were performed on sections of paraformaldehyde-fixed and paraffin-embedded tissues. Aortic root cross-sectional lesions (300 μm from beginning of aortic root) were stained with anti-CD68 (Abcam, ab5694) and smooth muscle actin (Abcam, ab125212) and Cy-3 labeled secondary antibodies. The analysis of positively stained areas was done blinded by two investigators with the ImageJ analysis software.

RNA analysis

Total RNA was isolated from homogenized tissue and cells and purified using E.Z.N.A. Hp Total RNA kit (Omega Tek) or RNeasy Mini kit (Qiagen) according to the manufacturer's instructions. The quality and quantity of the total RNA was monitored and measured with NanoDrop (NanoDrop Technologies, Inc. Wilmington, DE). For quantitative PCR analysis, 5-10 ng of cDNA was used for real-time PCR with gene-specific primers (Supplemental Table 2) and TBP as a house keeping gene on a BioRad CFX96 Real-time PCR system (Bio Rad).

RNA sequencing library preparation

Transcriptomes of quiescent BMDM (PBS) were compared to BMDM incubated with either 3'SL or LPS alone and LPS and 3'SL together. RNA was extracted from cells and stranded RNA-Seq libraries were prepared from polyA enriched mRNA using the TruSeq Stranded mRNA library prep kit (Illumina). Library construction and sequencing was performed by the University of California San Diego (UCSD) Institute for Genomic Medicine. Libraries were single-end sequenced for 76 cycles on a HiSeq 4000 to a depth of 20-30 million reads. For the Pam3CSK4 RNASeq at the Botnar Institute at Oxford University libraries were prepared using NEBNext

Poly(A) mRNA Magnetic Isolation Module from the NEBNext ultra II-directional RNA kit to create a first stranded library. Sequencing was then performed paired end 48 bp on a NextSeq 500 Illumina machine to 20-30 million reads.

Sequencing Data Analysis

FASTQ files from sequencing experiments were mapped to the mm10 assembly of the mouse genome. STAR with default parameters was used to map RNA-Seq experiments(4). Bowtie2 with default parameters was used to map ChIP-seq experiments(5). HOMER was used to convert aligned reads into “tag directories” for further analysis(6). Each sequencing experiment was normalized to total of 107 uniquely mapped tags by adjusting the number of tags at each position in the genome to the correct fractional amount given the total tags mapped. Sequence experiments were visualized by preparing custom tracks for the UCSC genome browser (7) using pooled tag directories. Peaks from ChIP-Seq and ATAC-Seq were defined using findPeaks command with default criteria (4-fold enrichment over background tag count; poisson enrichment p-value < 0.0001 over background tag count) as specified in HOMER documentation. Where applicable, IDR(8) was used to test for reproducibility between replicate experiments, and only peaks with IDR < 0.05 were used for downstream analysis. To quantify transcription factor binding and H3k27Ac at ATAC-defined accessible regions, peak files were merged with HOMER’s mergePeaks and annotated with raw tag counts using HOMER’s annotatePeaks using parameters -noadj, -size as indicated. Subsequently, DESeq2 (9) was used to identify the differential H3K27ac signal or chromatin accessibility with FC > 1.5. To identify motifs enriched in peak regions over the background, HOMER’s de novo motif analysis (findMotifsGenome.pl) including known default motifs and de novo motifs was used. The background sequences were either from random genome sequences or from peaks from the comparing condition indicated in the main text and in the figure legends. For RNA seq, each experiment was quantified using the

“analyzeRepeats” script of HOMER. To generate a table of raw read counts, the parameters -count exons -condenseGenes -noadj were used. To generate a table of TPM values, the parameters -count exons -condenseGenes -tpm were used. The TPM values were further processed by log2(TPM+1). DE genes were identified using raw sequencing read counts by DESeq2 (9) analysis through the “getDifferentialExpression” HOMER command at p-adj (adjusted p value) < 0.05 and FC (fold change) > 1.5. Metascape was used for gene ontology analysis(10).

Ingenuity Pathways Analysis (IPA) upstream regulator and canonical pathway analysis

Upstream regulators and canonical pathways for LPS vs. LPS+3’SL, PBS vs. 3’SL, PBS vs. LPS+3’SL, and PBS vs. LPS were generated utilizing Ingenuity Pathway Analysis (IPA, QIAGEN Inc., https://www.qiagenbioinformatics.com/products/ingenuitypathway-analysis). Specifically, after Benjamini-Hochberg FDR correction, genes with adjusted p-values less than 0.05 and fold change greater than 1.5 were considered as differentially expressed genes (DEGs). For example, the list of 134 identified DEGs for LPS vs. LPS+3’SL generated a total of 180 predicted significantly (|Z-score|>2) upstream regulators through IPA. The analysis was restricted to three molecule types: ‘G-protein coupled receptor’, ‘cytokine’, and ‘other’, and dysregulated canonical pathways were deemed significant when reaching a |Z-score| >2. The same procedures were applied to the comparisons of PBS vs. 3’SL, PBS vs. LPS+3’SL, and PBS vs. LPS.

Lipid and lipoprotein analysis

Lipid levels were analyzed in plasma and liver samples as previously described(11, 12). Blood was drawn via the tail vein from mice fasted for 4-5 hr. Total plasma cholesterol and plasma triglyceride levels (Sekisui Diagnostics) and NEFA levels (WAKO Diagnostics) were determined using commercially available kits.

snRNAseq of atherosclerotic aortas

Atherosclerotic aortas were cut underneath iliac bifurcation and perfused slowly with PBS to remove blood cells. Subsequently, perivascular fat was removed using a microscope. Aortas were chopped and digested in an enzyme solution containing collagenase type I (3.6 g/L, Sigma), collagenase type XI (10 mg/L, Sigma), Hyaluronidase (15 mg/L, Sigma), and DNase I (Invitrogen) in RPMI 1640 with 5% FCS and 1% Penicillin/Streptomycin (Gibco) and incubated at 37°C for 50 min. Digested aortas were poured through a 70 µm cell strainer (Falcon). Three-till-ten aortas per group were pooled to create 2 pools per genotype. Nuclei were centrifuged (1300rpm, 4°C, 10 min) and resuspended in PBS + 0.04% BSA. $12x10^4$ sorted cells per group were used for the construction of a barcoded library using a Chromium Controller (10x Genomics). Libraries were sequenced on an Illumina NOVA-seq platform. Data were analyzed and visualized using Cell Ranger (10x Genomics) and Seurat v4.0 in R environment. Initially, cells were filtered using the following conditions: nFeature_RNA > 25, nFeature_RNA < 6000, and percent.mito < 20. Furthermore, the R package, doubletfinder was utilized to remove potential doublets with the 8.5% doublet formation rate assumption. For integration of four different samples, the R package harmony was used with the default setting. After application of multiple functions including *RunUMAP*, *FindNeighbors* and *FindClusters*, clusters were defined and annotated manually using the marker genes from the resolution 0.8 and PCA dimension 50 outcomes. Unsupervised clustering on the remaining 5,889 cells from H_2O samples and 5,615 cells from 3'-SL identified 18 different clusters. To do so, the functions including *FindAllMarkers* and *FindMarkers* were used. Specifically, macrophage subsets were identified by the expression as reported before (13, 14). For visualization of UMAP, *DimPlot* function was applied with the cell type definition. Cell proportion plot was generated with the annotated dataset using the *aggregate* command and the *ggplot* tool. To examine the expression of gene, the restoration of genes was conducted using

the R package, *alra*. Differential genes were used to identify associated pathways using the metascape tool.

Foam cell assay

Macrophages were loaded with 100µg/nl of acetylated LDL (acLDL; ThermoFisher) for 24 hours. Macrophage cholesterol ester content was quantitated by the Amplex Red Cholesterol Assay Kit (Life Science Technologies, San Diego) and visualized by Oil Red O staining.(3) Cholesterol ester content was calculated by subtracting free cholesterol from total cholesterol for each sample.

Chromatin immunoprecipitation with RNA-Seq (ChIP-Seq)

ChIP for H3K27ac was performed essentially as describe previously (15) with minor modifications. Briefly, 1x10E6 bone marrow derived macrophages (BMDMs) were fixed with 1% paraformaldehyde in PBS for 15 minutes at room temperature. Next, 2.625 M glycine was added to 125 mM to quench fixation and cells were collected by centrifugation with at 1,100 X G for 10 minutes at 4C. Cells were washed twice with cold PBS and collected by centrifugation at 1,100 X G for 10 minutes at 4C. Cell pellets were then snap frozen and stored at -80C. For ChIP reactions, cell pellets were resuspended in 500 µl swelling buffer (10 mM HEPES/KOH pH7.9, 85 mM KCl, 1 mM EDTA, 0.5% IGEPAL CA-630, 1x protease inhibitor cocktail, 1 mM PMSF) and incubated on ice for 5 minutes. Cells were spun down and lysed in 500 µl LB3 (10 mM Tris/HCl pH 7.5, 100 mM NaCl, 1 mM EDTA, 0.5mM EGTA, 0.1% deoxcycholate, 0.5% sarkosyl, 1x protease inhibitor cocktail (Sigma), 1 mM PMSF). Chromatin was sheared to an average DNA size of 100-400 bp by administering 6 pulses of 10 seconds duration at 12 W power output with 30 seconds pause on wet ice using a Misonix 3000 sonicator. Samples were collected and 10% Triton X-100 was added to 1% final concentration. From each sample, 1% was taken as input DNA. For chromatin

immunoprecipitation, 30 µl of a 50:50 mixture of Dynabeads Protein A and Dynabeads Protein G coated with anti-H3K27ac (Active Motif) were incubated with slow rotation at 4°C overnight. For preparation of Dynabead-antibody complex, Dynabeads Protein A:G mixture and 4 µg specific antibody were incubated in 0.5% BSA/PBS for 1 hr at 4°C on rotator, then washed twice with 0.5% BSA/PBS and brought up to volume of 20 µl per IP with 0.1% BSA/PBS. After overnight incubation, the beads were collected using a magnet and washed three times each with wash buffer I (20 mM Tris/HCl pH 7.5, 150 mM NaCl, 1% Triton X-100, 0.1% SDS, 2 mM EDTA, 1x protease inhibitor cocktail, 1 mM PMSF) and wash buffer III (10 mM Tris/HCl pH 7.5, 250 mM LiCl, 1% Triton X-100, 0.7% Deoxycholate, 1 mM EDTA, 1x protease inhibitor cocktail, 1 mM PMSF). Beads were then washed twice with ice cold TE plus 0.2% Tween-20., then once with TE plus 50mM NaCl. Sequencing libraries were prepared for ChIP products while bound to the Dynabeads Protein A:G initially suspended in 25 µl 10 mM Tris/HCl pH 8.0 and 0.05% Tween-20. For LXR, SREBP, p65 and P300, ChIP-Seq with BMDMs was performed as described previously (Oishi et al., 2016, Sakai et al., 2020).

ChIP-Seq Library preparation

ChIP libraries were prepared while bound to Dynabeads using NEBNext Ultra II DNA Library preparation kit as previously described(16). Libraries were eluted and crosslinks reversed by adding to the 46.5 µl NEB reaction 20 µl water, 4 µl 10% SDS, 4.5 µl 5M NaCl, 3 µl 0.5 M EDTA, and 1 µl 20 mg/ml proteinase K, followed by incubation at 55°C for 1 hour and 65°C for 30 minutes to overnight in a thermal cycler. Dynabeads were removed from the library using a magnet and libraries cleaned by adding 2 µl SpeedBeads 3 EDAC in 61 µl 20% PEG 8000/1.5 M NaCl, mixing well, then incubating at room temperature for 10 minutes. SpeedBeads were collected on a magnet and washed two times with 150 µl 80% ethanol for 30 seconds. Beads were collected and ethanol removed following each wash. After the second ethanol wash, beads were air dried

and DNA eluted in 13 µl 10 mM Tris/HCl pH 8.0 and 0.05% Tween-20. DNA was amplified by PCR for 14 cycles in a 25 µl reaction volume using NEBNext Ultra II PCR master mix and 0.5 µM each Solexa 1GA and Solexa 1GB primers. PCR amplified libraries were size selected 200-500 bp using gel extraction using 10% TBE acrylamide gels. Libraries were single-end sequenced using either a HiSeq 4000 or a NextSeq 500 to a depth of 10-20 million reads.

EXTENDED DATA TABLES

Extended Data Table 1: Individually purchased human milk oligosaccharides.

Individual HMO	Company
**3’sialyllactose sodium salt (3’SL)**	GeneChem
**6’sialyllactose (6’SL)**	Kyowa
**2’fucosyllactose (2’FL)**	Jennewein Biotechnologie
**Disialyllacto-N-tetraose (DSLNT)**	V-labs, INC
**Lacto-N-fucopentaose 1 (LNFP-1)**	V-labs, INC

Extended Data Table 2: Primers used for qPCR analysis.

Gene	Forward primer (5'-3')	Reverse primer (5'-3')
CCL2, human	TCTCAAACTGAAGCTCGCACTC	GCATTGATTGCATCTGGCTGAG
Ccl2, mouse	AGGTCCCTGTCATGCTTCTG	GCTGCTGGTGATCCTCTTGT
Cxcl3, murine	ATACTGAAGAGCGGCAAGTCC	AGACACCGTTGGGATGGATC
Fdps, murine	CTGGTGGTGCCAAGTGTGGAC	GAAGTGCTGGATGAAATTCTGC
ICAM, human	GGAGCACTCAAGGGGAGGTC	TGGCGGTTATAGAGGTACGTGC
Il10, murine	TGAATTCCCTGGGTGAGAAG	TCACTCTTCACCTGCTCCACT
IL1b, human	GTGGCAATGAGGATGACTTGTTC	TAGTGGTGGTCGGAGATTCGTA
Il1b, murine	AAATACCTGTGGCCTTGGGC	CTTGGGATCCACACTCTCCAG
IL6, human	AGCCACTCACCTCTTCAGAAC	GCCTCTTTGCTGCTTTCACAC
Il6, murine	CCAGAGATACAAAGAAATGATGG	ACTCCAGAAGACCAGAGGAAAT
IL8, human	AGCTGGCCGTGGCTCTCTTG	GGGTGGAAAGGTTTGGAGTATG
LRP8, human	GAAGCGGAACTATTCACGCCTC	ATGAGGACCTCCTGCTCTTTCG
Lrp8, murine	AAGATTGAGAAGGCTGGGCTC	TACAAACGCTGGCTCAGCAAG
Ptgs2, murine	TGCACTATGGTTACAAAAGCTGG	CAGTCCGGGTACAGTCACAC
Pparg, murine	GCATGGTGCCTTCGCTGA	TGGCATCTCTGTGTCAACCATG
Saa3, murine	GTCCAGTTCATGAAAGAAGCTG	AGCATGGAAGTATTTGTCTGAG
Stard4, murine	CACAGCATCGAAGAAGATGAG	GTCATCCATAACTCCTTGAGC
SCD5, human	GGTGGCTGTTTGTTCGCAAG	ACCACAAAGCACATGAGCAC
SQL, human	CTCCAAGTTCAGGAAAAGCCTGG	GAGAACTGGACTCGGGTTAGCT
TBP, human	CACGAACCACGGCACTGATT	TTTTCTTGCTGCTGCCAGTCTGGAC
Tbp, murine	GAAGCTGCGGTACAATTCCAG	CCCCTTGTACCCTTCACCAAT
TNF, human	GCCCTGTGAGGAGGACGAAC	AAGAGGTTGAGGGTGTCTGAAGGA
Tnf, murine	ATTCGAGTGACAAGCCTGTAGC	GGTTGTCTTTGAGATCCATGCC
VCAM, human	GCGGGAGTATATGAATGTGAATC	AAGGAGGATGCAAAATAGAGCAC

EXTENDED DATA FIGURES AND LEGENDS

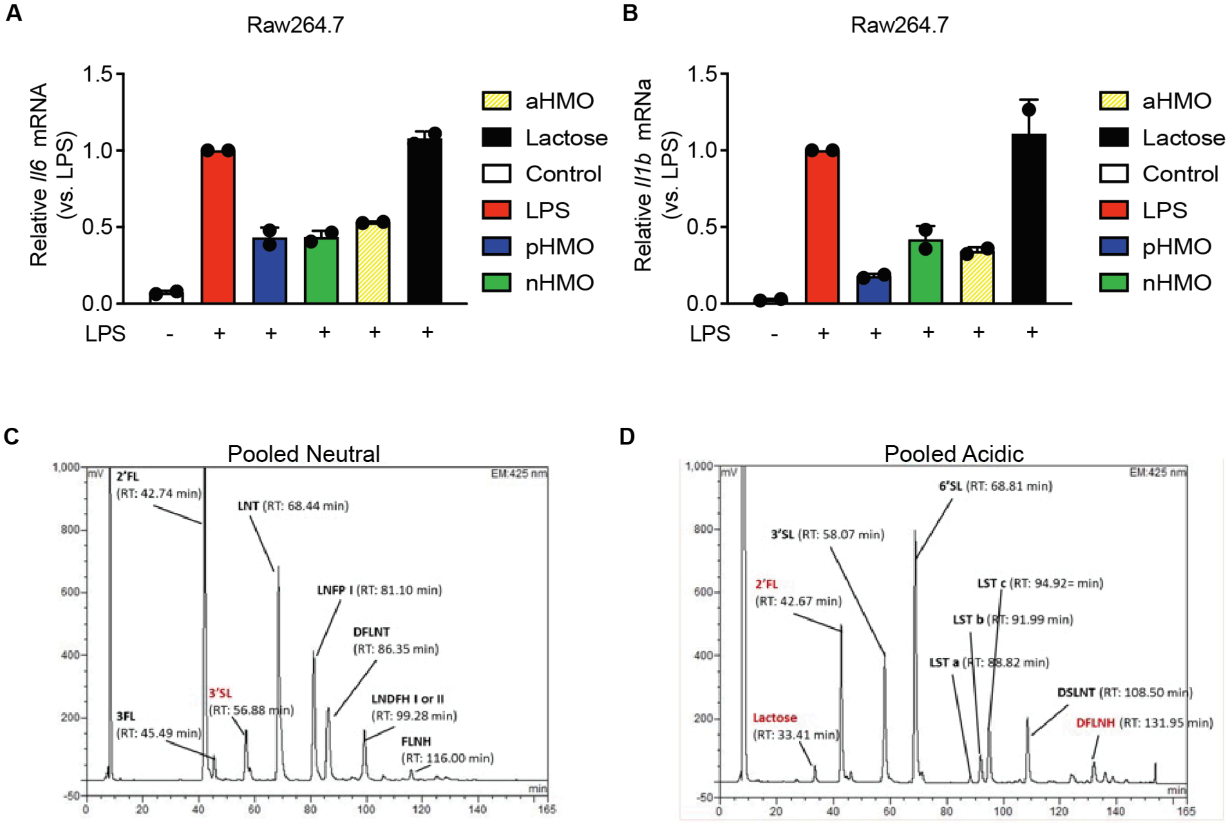

Extended Data Figure 1. A-B, Relative expression of *Il6* (**A**) *and Il1β* (**B**) in Raw264.7 cells when treated with LPS + pHMO, neutral (nHMO), acidic (aHMO), and lactose (all 500 µg/mL) (n = 2). **C-D**, Representative HPLC spectra of pooled (**D**) nHMO and (**D**) aHMO fractions. Statistical analysis with one-way ANOVA vs. LPS with Dunnett's post hoc test. (* $p<0.05$; ** $p<0.01$; *** $p<0.001$); Bar plots represent mean ± SEM.

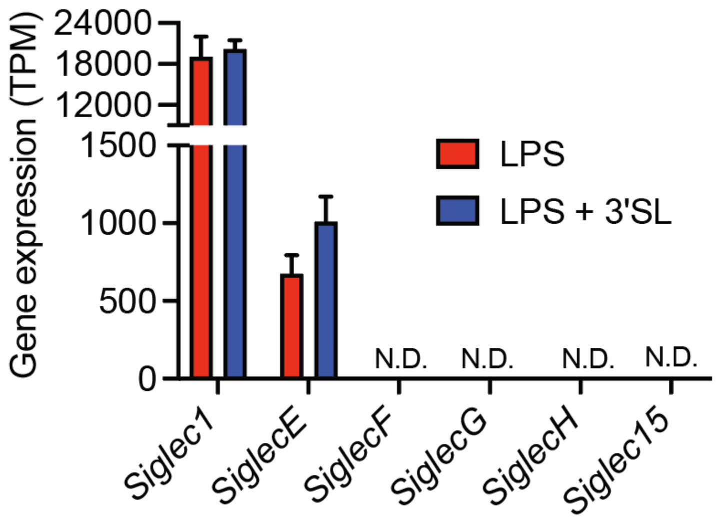

Extended Data Figure 2. Siglec expression determined by RNA-seq of BMDMs treated with (10 ng/ml) LPS or LPS + 3'SL (100μg/ml) (n = 3/group).; Bar plots represent mean ± SEM.

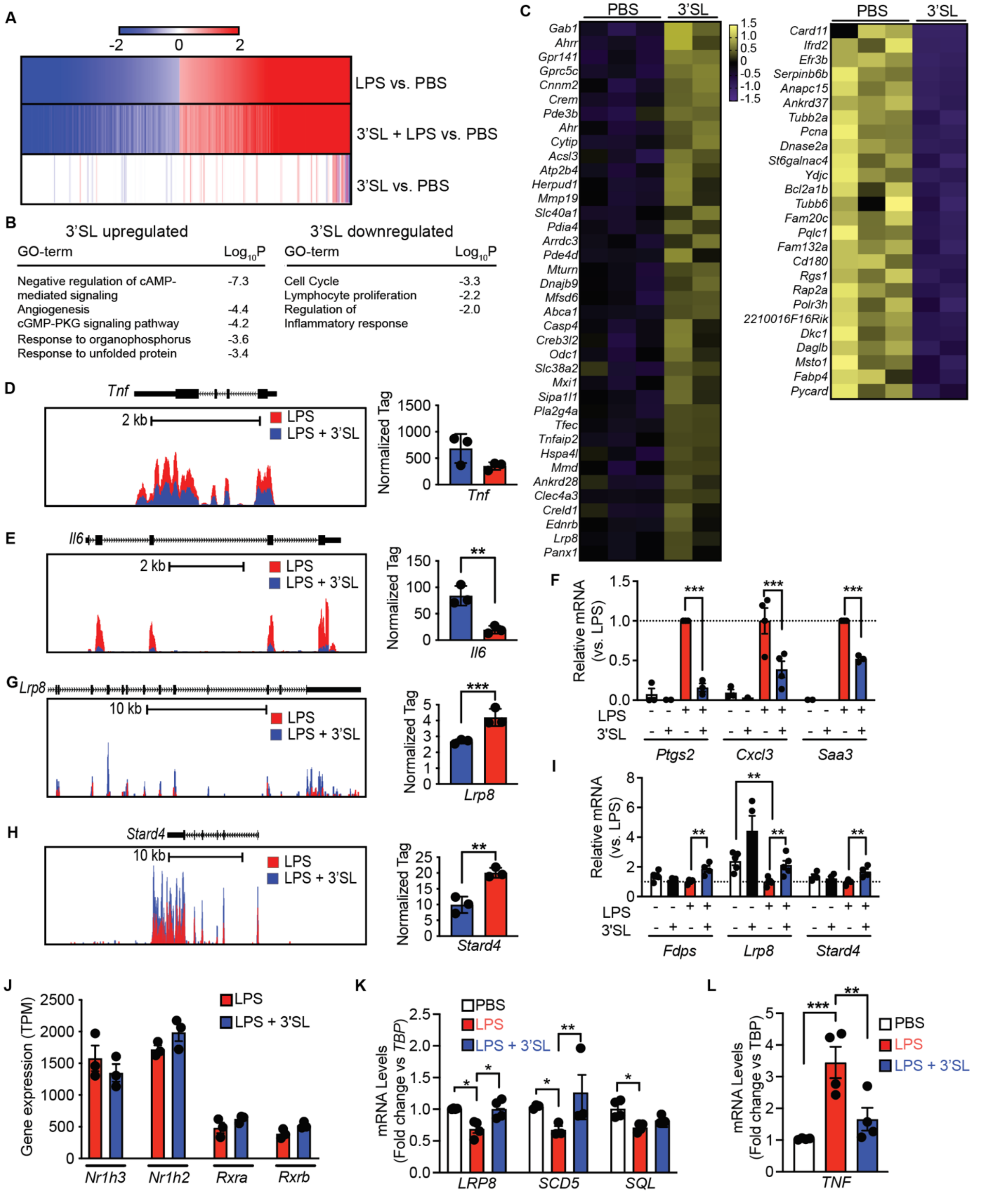

Extended Data Figure 3. A, Heat map representing z-normalized row expression of each gene for RNA-seq of all significantly up-regulated (red) or down-regulated (blue) genes in BMDMs at 6 h after 3'SL or LPS ± 3'SL stimulations in comparison with quiescent BMDMs (n = 3). **B**, Pathway

analyses of 3'SL down-regulated and up-regulated genes in quiescent BMDMs. **C**, Heat maps representing fold-change expression of up-regulated (yellow) or down-regulated (blue) genes in quiescent BMDMs at 6 h after 3'SL stimulation (cut-off $p < 0.05$ and fold change, FC, >1.5) **D-E**, Normalized tag counts for *Tnf* and *Il6* with normalized tag count averages (n=3). **F**, qPCR verification of *Ptgs2*, *Cxcl3* and *Saa3* in BMDMs (n ≥ 3). **G-H**, Normalized tag counts for *Lrp8* and *Stard4* with normalized tag count averages (n = 3). **I**, qPCR verification of *Fdps*, *Lrp8* and *Stard4* in BMDMs (n ≥ 3). **J**, RNA-seq analysis of LXR and RXR gene expression (n = 3). **K-L** qPCR verification of *LRP8, SCD5, SQL* and *TNF* in human peripheral blood monocytes (hPBMCs) (n ≥ 3) (** $p<0.01$; *** $p<0.001$); Bar plots represent mean ± SEM.

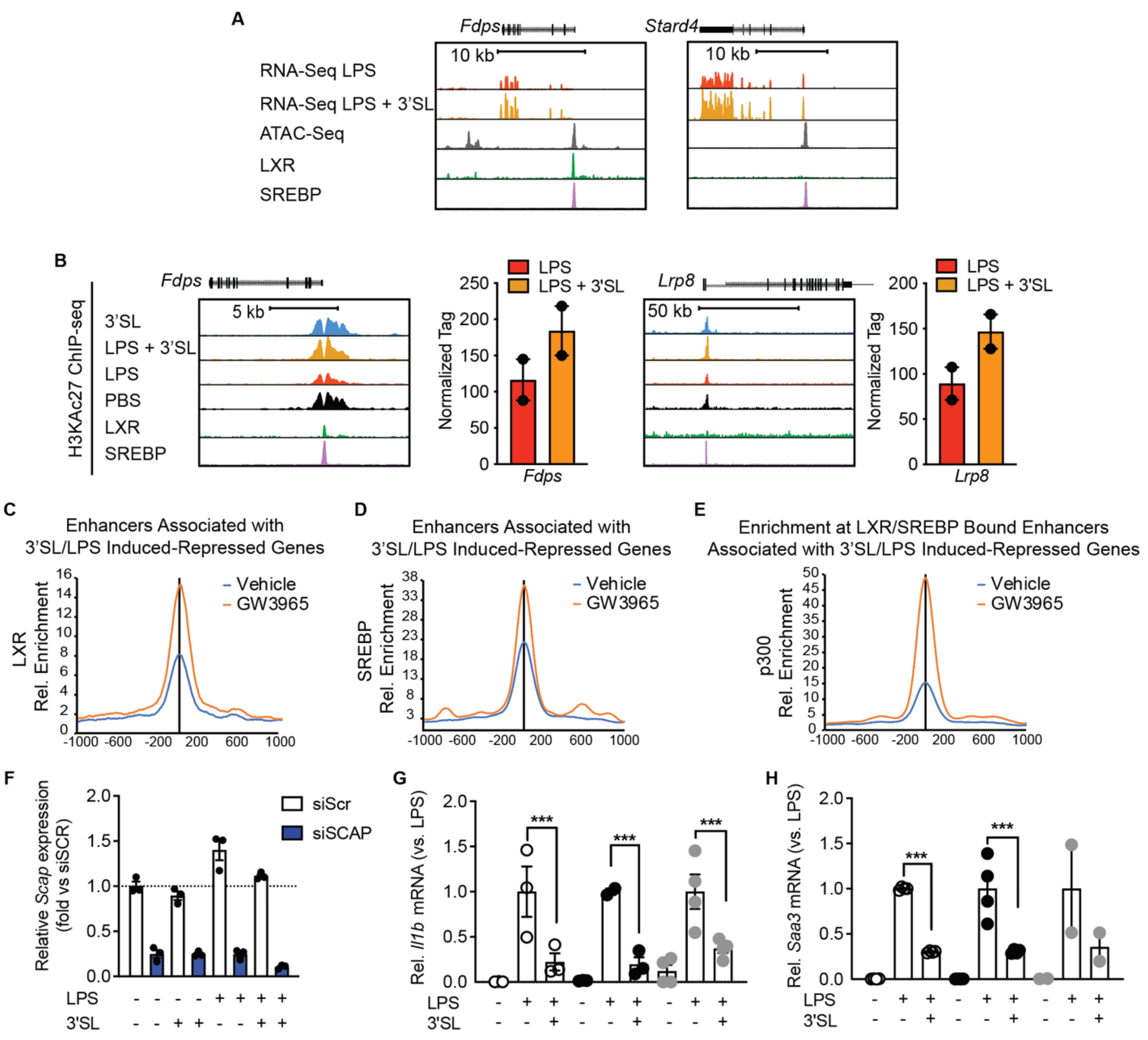

Extended Data Figure 4. A, UCSC genome browser images illustrating normalized tag counts for *Fdps* and *Stard4* and illustrating ATAC-seq as well as mapped LXR and SREBP binding sites. **B**, UCSC genome browser images illustrating normalized tag counts for H3K27ac at 3'SL induced target loci together with mapped LXR and SREBP binding sites. **C-E**, Distribution of enhancers associated with 3'SL-LPS induced-repressed genes in the vicinity of genomic regions of vehicle and GW3965 treated macrophages co-bound by LXR (**C**) or SREBP (**D**) and p300 (**E**) enrichment at these sites. **F**, Relative *Scap* expression in BMDMs transfected with non-targeting control siRNA (siScr) or si*Scap* (n = 4). **G**, Relative expression of target genes in BMDMs transfected

with non-targeting control siRNA (siScr) or si*Scap* or BMDMs isolated from *Lxrα/β* knock-out mice (*Lxr*$^{-/-}$) stimulated with PBS and LPS ± 3'SL. (n silencing experiments = 4, *Lxr*$^{-/-}$ BMDMs from 3 individual mice). (*p<0.05; ** p<0.01; *** p<0.001); Bar or dot plots represent mean ± SEM.

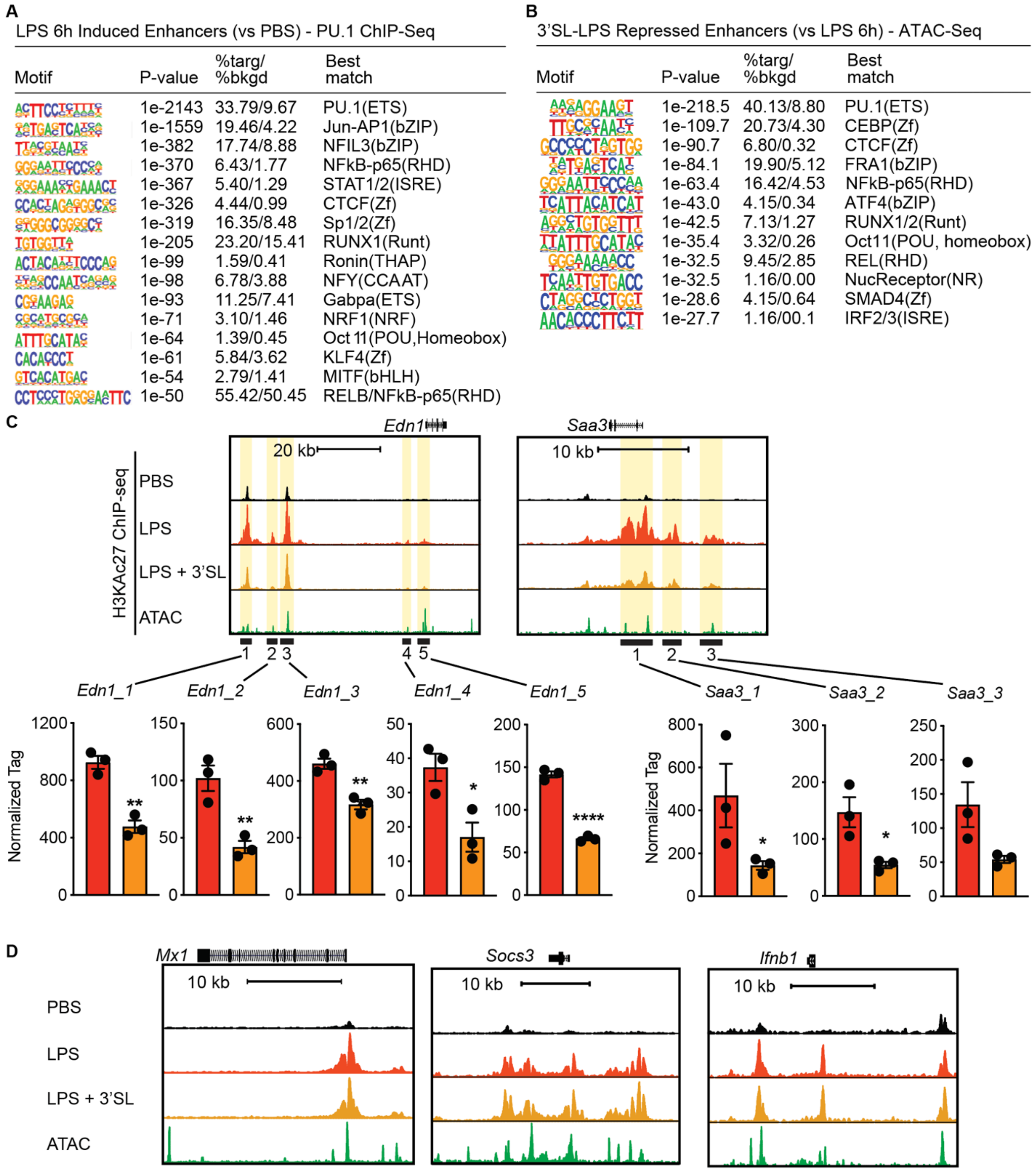

Extended Data Figure 5. A, *De novo* motif analysis of LPS upregulated enhancers (vs. LPS) using a GC-matched genomic background using ATAC-Seq and Pu.1 bound enhancers. **B**, *De novo* motif analysis of 3'SL-LPS repressed enhancers using a GC-matched genomic background on ATAC-Seq of LPS activated enhancers. **C,** UCSC genome browser images illustrating

normalized tag counts for H3K27ac and illustrating ATAC-seq peaks at 3’SL repressed target loci.

D, UCSC genome browser images illustrating normalized tag counts for H3K27ac and illustrating ATAC-seq peaks at target loci activated by LPS but unaffected by 3’SL.

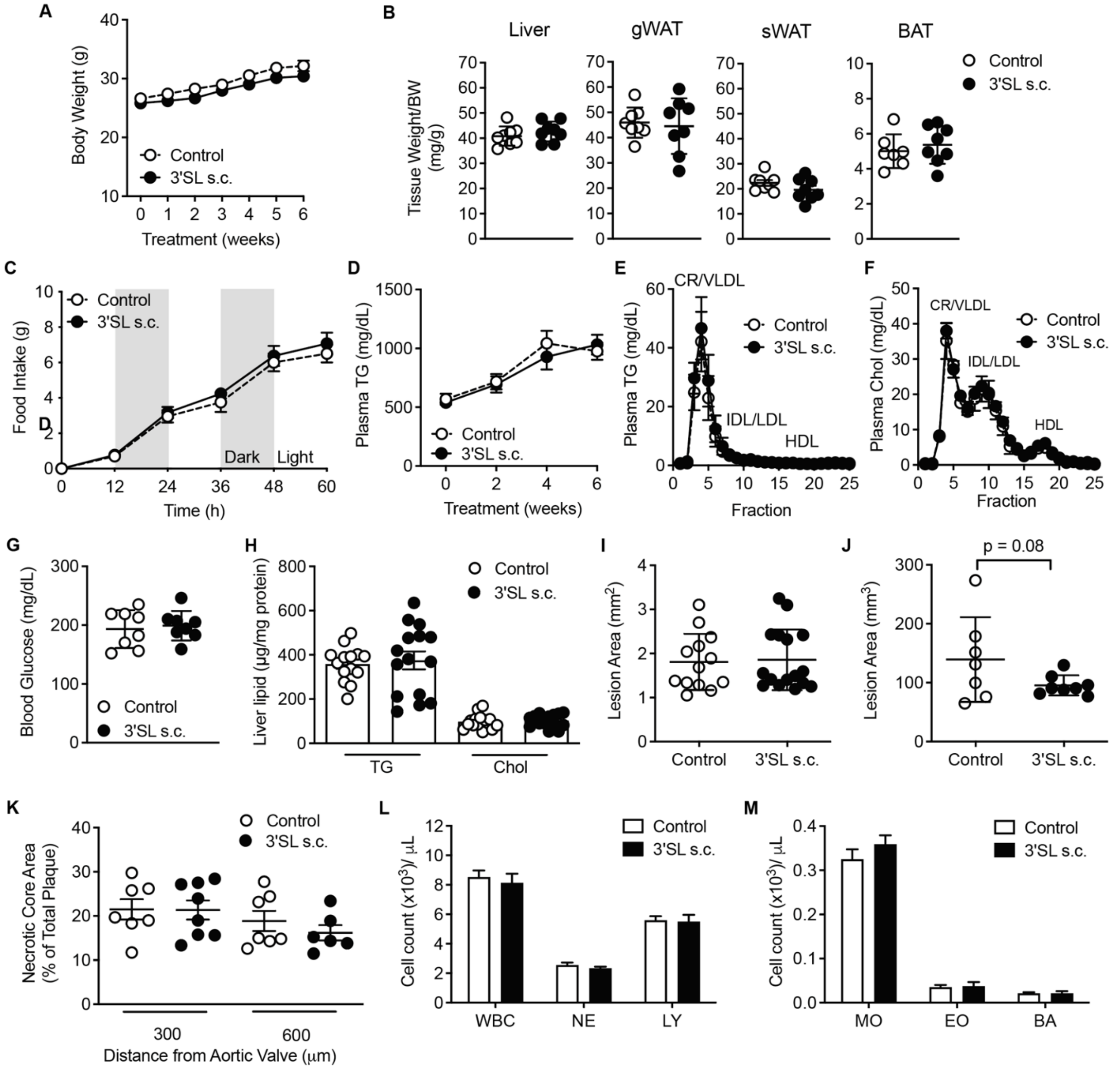

Extended Data Figure 6. A, Weekly body weight in subcutaneous (s.c.) 3'SL treated and control PBS treated *Ldlr*$^{-/-}$ mice (n = 8). **B**, Organ weights at harvest of Liver; gWAT, gonadal white adipose tissue; sWAT, subcutaneous WAT; BAT, brown adipose tissue (n = 8). **C**, Food intake over 60 hours measured every 12 hours. **D**, Bi-weekly plasma triglyceride (TG) levels in 3'SL treated and control *Ldlr*$^{-/-}$ mice (n = 14-15). **E-F**, FPLC TG and Cholesterol lipoprotein profiles after 6 weeks of treatment (two pooled samples per group of 7-8). **G**, Plasma glucose was measured from full blood after 4 weeks of treatment (n = 8). **H**, Liver lipid levels after 6 weeks of treatment

(n = 14-15). **I**, *En face* analysis of atherosclerosis and quantification of Sudan IV-positive area (n = 14-16). **J**, Average lesions size as determined by aortic root analysis (n = 7-8). **K**, Quantification of necrotic core size (n = 7-8). **L-M**, White blood cell counts (WBC) in blood after 4 weeks of treatment. NE, neutrophils; LY, lymphocytes; MO, monocytes; EO, eosinophils; BA, basophils. All plasma parameters were measured after indicated treatment periods following a 5-hour food restriction. Bar or dot plots represent mean ± SEM.

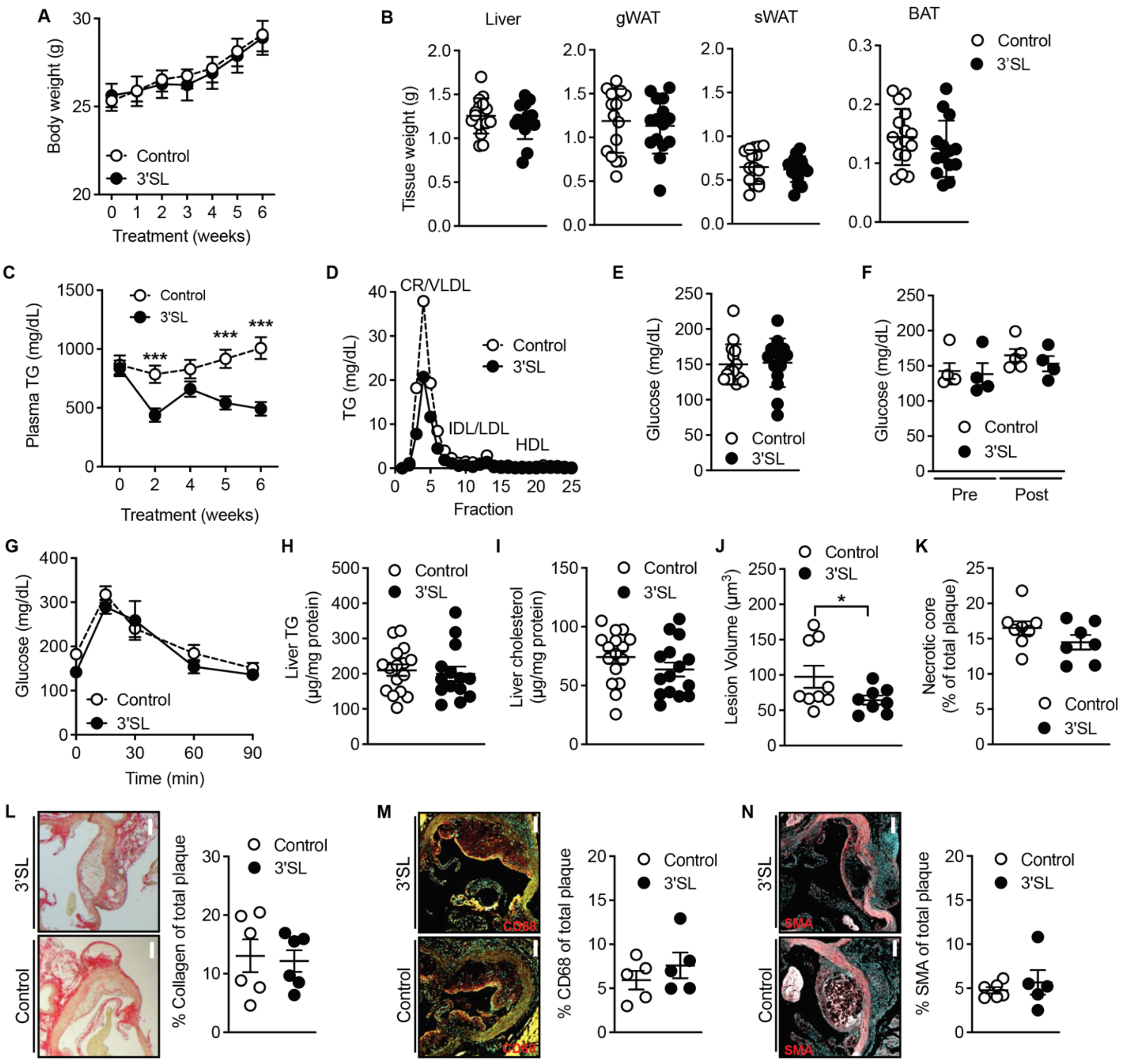

Extended Data Figure 7. A, Weekly body weight in oral 3'SL treated and control treated *Ldlr*$^{-/-}$ mice (n = 14). **B**, Organ weights at harvest of Liver; gWAT, gonadal white adipose tissue; sWAT, subcutaneous WAT; BAT, brown adipose tissue (n = 8). **C**, Bi-weekly plasma triglyceride (TG) levels in 3'SL treated and control *Ldlr*$^{-/-}$ mice (n = 14-15). **D**, FPLC TG lipoprotein profiles after 6 weeks of treatment (two pooled samples per group of 7-8). **E**, Plasma glucose was measured from full blood after 4 weeks of treatment (n = 8). **F**, Plasma glucose levels was measured after 4h fasting before and 20 min after gavaging a 90 mg bolus of 3'SL. **G**, Oral glucose tolerance test

after 4 weeks of treatment showed no differences in glucose handling. **H-I**, Liver lipid levels after 6 weeks of treatment (n = 14-15). **J**, Average lesions size as determined by aortic root analysis (n = 8-9). **K**, Quantification of necrotic core size (n = 7-8). **L**, Picrosirius red staining for collagen of equal sized atherosclerotic lesions and quantification of the positive stained area (n = 6). **M**, Atherosclerotic lesions stained with CD68 for macrophages and quantification of the positive stained area (n = 5). **N**, Smooth muscle actin (SMA) staining and quantification of the positive stained area (n = 5-6). All plasma parameters were measured after indicated treatment periods following a 5-hour food restriction. (*** $p<0.001$); Bar or dot plots represent mean ± SEM.

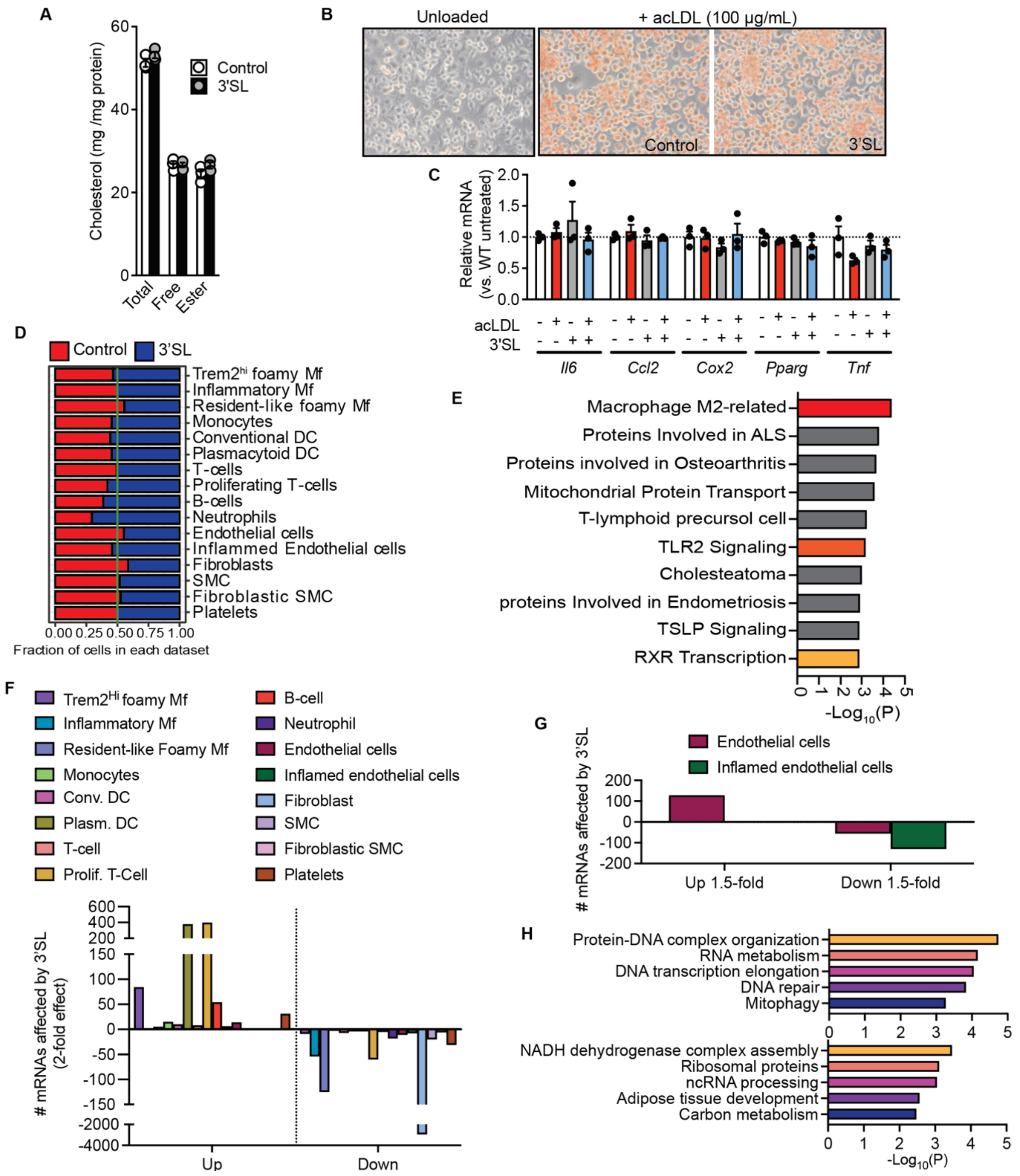

Extended Data Figure 8. A, Determination of total, esterified and unesterified cholesterol levels in BMDMs challenged with 100 µg/ml of acetylated LDL (acLDL) for 24 hrs. co-treated with or without 3'SL (100 µg/ml) (n=4/group). **B**, Representative micro-images from BMDMs stained with Oil red O. **C**, qPCR expression analysis of the indicated chemokines in BMDMs challenged with 100 µg/ml of acetylated LDL (acLDL) for 24 hrs, co-treated with or without 3'SL (100 µg/ml)

(n=4/group). **D**, Relative distribution of atherosclerotic lesion cell subsets in control vs 3'SL treated mice from the snRNA-seq. **E**, Enriched pathways identified by metascape analysis software of the mRNA transcripts that are differentially expressed upon in myeloid cells upon 3'SL treatment. **F**, The number of significantly differentially expressed mRNA gene transcripts in the indicated cell subsets and their directionality upon 3'SL administration (up is >2-fold; down is <-2-fold; compared to control treatment) **G**, The number of significantly differentially expressed mRNA gene transcripts in the indicated endothelial cell subsets and their directionality upon 3'SL administration (up is >1.5-fold; down is <-1.5-fold; compared to control treatment). **H**, Enriched pathways identified by metascape analysis software of differentially expressed mRNA in macrophage subsets upon 3'SL administration (>1.5-fold or <-1.5 change; compared to control treatment). Plots represent mean ± SEM.

References

1. Kunz C, et al. High-pH anion-exchange chromatography with pulsed amperometric detection and molar response factors of human milk oligosaccharides. *J Chromatogr B Biomed Appl.* 1996;685(2):211-21.
2. Repa JJ, et al. Regulation of absorption and ABC1-mediated efflux of cholesterol by RXR heterodimers. *Science.* 2000;289(5484):1524-9.
3. Gordts P, et al. Reducing macrophage proteoglycan sulfation increases atherosclerosis and obesity through enhanced type I interferon signaling. *Cell Metab.* 2014;20(5):813-26.
4. Dobin A, et al. STAR: Ultrafast universal RNA-seq aligner. *Bioinformatics.* 2013;29:15-21.
5. Langmead B, et al. Fast gapped-read alignment with Bowtie 2. *Nat Methods.* 2012;9:357-9.
6. Heinz S, et al. Simple combinations of lineage-determining transcription factors prime cis-regulatory elements required for macrophage and B cell identities. *Mol Cell.* 2010;38(4):576-89.
7. Kent WJ, et al. The human genome browser at UCSC. *Genome Res.* 2002;12(6):996-1006.
8. Li Q, et al. Measuring reproducibility of high-throughput experiments. *The Annals of Applied Statistics.* 2011;5(3):1752-79.
9. Love MI, et al. Moderated estimation of fold change and dispersion for RNA-seq data with DESeq2. *Genome Biol.* 2014;15(12):550.
10. Zhou Y, et al. Metascape provides a biologist-oriented resource for the analysis of systems-level datasets. *Nat Commun.* 2019;10(1):1523.
11. Gordts PL, et al. ApoC-III inhibits clearance of triglyceride-rich lipoproteins through LDL family receptors. *J Clin Invest.* 2016;126(8):2855-66.

12. Ramms B, et al. ApoC-III ASO promotes tissue LPL activity in the absence of apoE-mediated TRL clearance. *J Lipid Res.* 2019;60(8):1379-95.

13. Willemsen L, et al. Macrophage subsets in atherosclerosis as defined by single-cell technologies. *J Pathol.* 2020;250(5):705-14.

14. Willemsen L, et al. DOT1L regulates lipid biosynthesis and inflammatory responses in macrophages and promotes atherosclerotic plaque stability. *Cell Rep.* 2022;41(8):111703.

15. Eichenfield DZ, et al. Tissue damage drives co-localization of NF-κB, Smad3, and Nrf2 to direct Rev-erb sensitive wound repair in mouse macrophages. *eLife.* 2016;5:554-62.

16. Heinz S, et al. Transcription Elongation Can Affect Genome 3D Structure. *Cell.* 2018;174(6):1522-36 e22.
