# Supplementary figures and images for: The human milk oligosaccharide 3′sialyllactose reduces low-grade inflammation and atherosclerosis development in mice
Source: JCI Insight. 2024 Nov 8;9(21):e181329. doi: 10.1172/jci.insight.181329 (PMC11601559; doi:10.1172/jci.insight.181329)

UNCROPPED gels for Fig. 2C

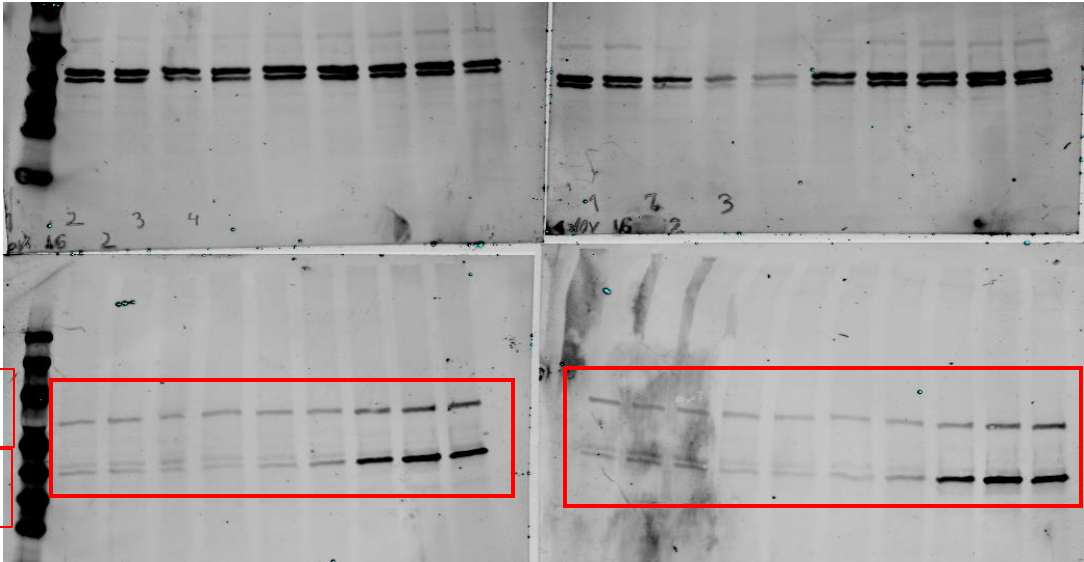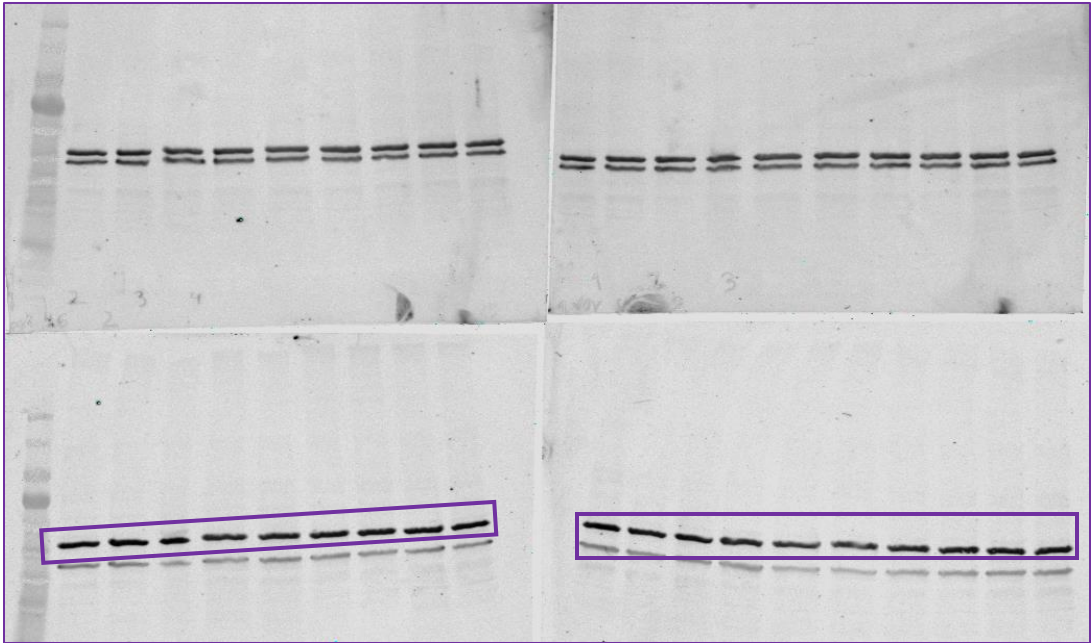

Supplement: Unedited blot and gel images [file jciinsight-9-181329-s140.pdf]
